# AcWRKY75 and AcMYB108 mediated ABA-GA antagonism in the regulation of pineapple internal browning

**DOI:** 10.1186/s43897-026-00247-6

**Published:** 2026-10-11

**Authors:** Qian Li, Guang Wang, Shijiang Zhu

**Affiliations:** 1 Guangdong Province Key Laboratory of Postharvest Physiology and Technology of Fruit and Vegetables, College of Horticulture, South China Agricultural University, 483 Wushan Road, Tianhe District, Guangzhou, 510642 China; 2 College of Horticulture, Qingdao Agricultural University, No. 700 Changcheng Road, Chengyang, Qingdao City, Shandong Province 266109 China

**Keywords:** Pineapple, Internal browning, Phenolic biosynthesis, Transcriptional regulation, Abscisic acid, Gibberellic acid

## Abstract

Gibberellic acid (GA) and abscisic acid (ABA) antagonistically regulate various biological processes, but whether they antagonistically regulate tissue browning remains unclear. Here, we showed that ABA reduced pineapple internal browning (IB) by inhibiting phenolic biosynthesis via downregulating AcWRKY75 and AcMYB108, while GA_3_ had the opposite effects. Biochemical evidences showed AcWRKY75 and AcMYB108 directly bound to and activated the phenolic biosynthetic gene *Ac4CL5*. Interestingly, AcMYB108 directly bound to *AcWRKY75* promoter and activated its transcription, indicating that AcMYB108 functions upstream of *AcWRKY75. Arabidopsis* overexpressing *AcWRKY75*, *AcMYB108* or *Ac4CL5* displayed promoted phenolic biosynthesis. *AcWRKY75* transient overexpression in pineapple reduced, while its transient silencing enhanced IB and phenolic accumulation. Transcriptomic profiling of pineapples transiently overexpressing and silencing *AcWRKY75* revealed that plant hormone signal transduction and phenylpropanoid biosynthesis were among the most significantly enriched KEGG pathways, suggesting the role of AcWRKY75 in regulating phenolic biosynthesis involves plant hormones. ABA suppressed and GA_3_ enhanced enzyme activity of GUS transiently expressed in tobacco driven by promoter of *AcWRKY75*, *AcMYB108* or *Ac4CL5*. This work suggests that WRKY75 and MYB108 are involved in antagonism between ABA and GA in regulating IB, and may shed novel insight into the role of ABA and GA in regulating plant tissue browning.

**Supplementary Information:**

The online version contains supplementary material available at https://doi.org/10.1186/s43897-026-00247-6.

## Core

Internal browning (IB) affects pineapple quality. Here we show WRKY75 and MYB108 involved in antagonism between ABA and GA in regulating IB, which may help breed IB-free pineapples and shed light on role of ABA and GA.

## Gene & accession numbers

Sequence data from this article can be found in the NCBI data libraries under accession numbers: *AcWRKY75* (LOC109726264), *AcMYB108* (LOC109718059), *AcC4H* (LOC109706879), *Ac4CL* (LOC109711909), *AcHCT* (LOC109706912), *AcC3H* (LOC109728123), *AcCSE* (LOC1097112336), *AcActin* (GenBank: HQ148720.1).

## Introduction

Abscisic acid (ABA) and gibberellin (GAs) are antagonistic in regulating a series of biological processes, such as seed dormancy and germination (Shu et al. 2016), bud break (Singh et al. 2018), plant growth and development (Shu et al. 2018) and plant stress responses (Liu and Hou 2018). Phenolic compounds constitute an important category of secondary metabolites with diversified functions in biological processes in plants (Fang et al. 2022). For instance, they substantially function in bud dormancy of sweet cherry buds (Baldermann et al. 2018) and seed germination of germinated cereal and pulse seeds (Xu et al. 2020). In addition, the accumulation of phenolic compound is associated with tissue browning caused by senescence (Lorente-Mento et al. 2023) and responses to stresses, e. g. chilling injury (Kuang et al. 2023), loss of water (Lorente-Mento et al. 2023) and diseases (Liu et al. 2018). Whether and how the antagonism between ABA and GA in regulating biological processes involves phenolic biosynthesis remains poorly understood. Tissue browning is a common phenomenon happening in plants, especially during senescence (Zhai et al. 2018) and responses to abiotic (Iakimova and Woltering 2018) and biotic stresses (Hayashi et al. 2016). The occurrence of tissue browning may severely affect plant growth and development (Tang and Newton 2004) and crop quality (Anastasiadi et al. 2021). Tissue browning is closely associated with accumulation of phenolic compounds in pineapple (Van Lelyveld and De Bruyn 1976; Li et al. 2023c) and apple (Fang et al. 2022). Phenolic compounds are produced by phenylpropanoid pathway, which starts with phenylalanine ammonia lyase (PAL) converting phenylalanine to trans-cinnamic acid, and the latter is further converted to p-coumaroyl CoA by cinnamate 4-hydroxylase (C4H) and 4-coumarate: coenzyme A (CoA) ligase (4CL) (Ma et al. 2018), which thus provides the precursors for down-stream branches to produce a variety of metabolites, including lignin, flavonoids, and phenolics (Tan et al. 2023). Many transcription factors (TFs) are involved in modulating biological processes that are regulated by ABA and GAs. The MYB and WRKY TF families are among the most important. WRKY belongs to a large family of TFs, containing WRKYGQK conserved amino acid sequences and two zinc-finger-like motifs at the C-terminal, C-X 4–5 -C-X 22–23 -H–X-H (C2H2) and CX 7 -C-X 23 -H–X-C (C2HC) (Eulgem et al. 2000). OsWRKY51 and OsWRKY71, which are ABA-inducible and GA-repressible, connect the ABA and GA signaling in aleurone cells of rice (Xie et al. 2006). MdWRKY31 positively regulates sensitivity of apple to ABA (Zhao et al. 2019), while OsWRKY5 negatively regulates tolerance to drought induced by ABA (Lim et al. 2022). GA suppressed leaf senescence by reducing expression of *BrWRKY6* in Chinese flowering cabbage (Fan et al. 2018). The MYB TFs form the second largest TF family in eukaryotes and play critical roles in diversified biological processes (Wang et al. 2023). *PpMYB52*, a negative regulator for bud break of peach, is suppressed by GA and induced by ABA (Zhang et al. 2022)*.* ABA promotes the expression of *FaMYB10*, resulting in anthocyanin accumulation (Kadomura-Ishikawa et al. 2015). GA promotes cell division and root growth by inhibiting biosynthesis of flavonol through reducing the transcriptional activity of *MYB12* (Tan et al. 2019)*.* GA_3_ promotes lignin accumulation of walnut (*Juglans regia* L.) fruit by inducing *JrMYB180* expression (Li et al. 2023a). Pineapple internal browning (IB) is an important physiological disorder usually occurring in fruit during postharvest processes (Li et al. 2023c). Pineapple can be grouped into two categories. One, represented by the cultivar ‘smooth cayenne’, develops IB under low temperature (10 °C or lower) storage (Paull and Rohrbach 1985; Setiawan et al. 2024). While the other, represented by the cultivar ‘Comte de Paris’, shows IB at ambient temperature (AT, 20 °C or higher) (Zhang et al. 2015; Hong et al. 2023), but not at low temperature (LT, Hong et al. 2013; Zhang et al. 2016). ‘Comte de Paris’ is the leading pineapple cultivar in China. The pineapple industry in China suffers from great economic losses annually, as pineapples are usually transported and marketed under AT (Zhang et al. 2015). Therefore, deciphering the mechanism underlying IB development at AT and looking for ways to control IB are of scientific and economic significance.

Exogenous GAs enhances IB for both of the two categories (Pusittigul et al. 2012; Zhang et al. 2016; Li et al. 2023b), while ABA alleviates IB of ‘Comte de Paris’ pineapple stored at AT (Zhang et al. 2015; Li et al. 2023c), although no study has shown so far whether ABA treatment could reduce IB of ‘smooth cayenne’ pineapple caused by LT storage. For both ‘smooth cayenne’ (Soares et al. 2005) and ‘Comte de Paris’ (Li et al. 2023b), the IB fruit have higher total phenolic contents (TPC) than the healthy ones. This implies that ABA and GA may antagonize with each other in regulating phenolic accumulation in ‘Comte de Paris’. However, whether and how WRKY and MYB TFs are differentially regulated by ABA and GA to modulate phenolic biosynthesis remains elusive.

Previously we showed that AcbHLH144 TF functions as a repressor for phenolic biosynthesis and that ABA alleviates pineapple IB via enhancing it (Li et al. 2023c). Here in this study, two TFs AcWRKY75 and AcMYB108 were identified from pineapple fruit. Multiomics together with biochemical and genetic approaches were used to investigate whether these TFs were differentially modulated by ABA and GA in regulating phenolic biosynthesis. It is hoped that the findings could provide new insight into the antagonism between ABA and GA in regulating tissue browning-related biological processes in plants.

## Results

### ABA and GA_3_ differentially regulated pineapple IB and phenylpropanoid biosynthesis pathway

**Fig. 1:**
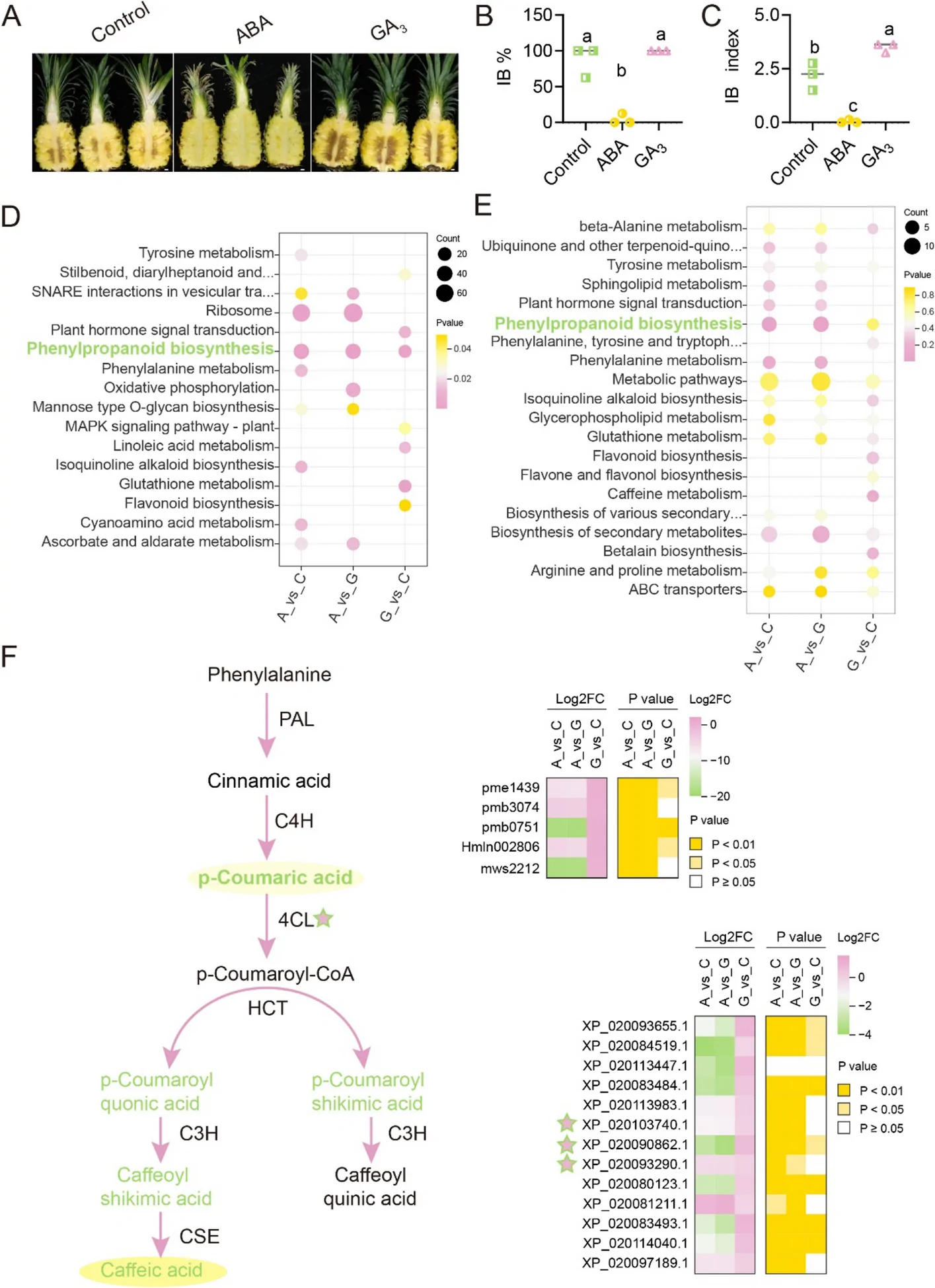
Exogenous GA_3_ and ABA differentially regulated phenylpropanoid biosynthesis in pineapple.** A** The IB symptoms in pineapple fruit stored for 9 d at 20 °C after treatment with ABA (200 mg L^−1^) or GA_3_ (200 mg L^−1^). Scale bar = 1 cm. **B** IB incidence of fruit was evaluated at 9 d after treatment. **C** IB index of fruit was evaluated at 9 d after treatment. **D** The top 16 KEGG pathway enrichment of differentially expressed proteins (DEPs) in pineapple. C, Control; A, ABA; G, GA_3_. **E** The top 20 KEGG pathway enrichment of differentially accumulated metabolites (DAMs) in pineapple. C, Control; A, ABA; G, GA_3_.** F** Reconstructed phenolic biosynthesis pathway with DAMs and DEPs (left). The heat maps showing the DAMs and DEPs, where each line represents a metabolite or a protein (right). The colors of charts represent upregulation (light purple) and downregulation (green). C, Control; A, ABA; G, GA_3_. Data are given as mean ± SD (n = 3) and significance of differences is indicated by letters above the bars (*P* < 0.05), unless otherwise indicated

Following 9-d storage at AT, the ABA-treated pineapples were much slighter in IB symptoms, and much lower in IB incidence and index than the Control fruit, while the GA_3_-treated fruit were more severe in symptoms and higher in IB index than the Control (Fig. 1 A-C). This indicates that ABA suppressed and GA_3_ aggravated IB. To investigate the mechanism of differential regulation of IB by the two phytohormones, proteomic and metabolomic analysis were conducted. As shown in Fig. 1D, 16 KEGG pathways were significantly enriched by differentially expressed proteins (DEPs) for ABA *vs* Control and ABA vs GA_3_, respectively. Among them, three pathways, i. e. phenylpropanoid biosynthesis, ascorbate and aldarate metabolism, and ribosome, were shared by ABA *vs* Control and ABA *vs* GA_3_ (Fig. 1D). Interestingly, only phenylpropanoid biosynthesis was shared by ABA *vs* Control and ABA *vs* GA_3_ in terms of the KEGG pathways significantly enriched by differentially accumulated metabolites (DAMs) (Fig. 1E). The proteomic and metabolomic data were used to reconstruct the phenolic biosynthesis pathway. As shown in Fig. 1F, compared with the control and GA_3_ treatment, ABA downregulated twelve of the thirteen DEPs for the six key enzymes involved in biosynthesis of phenolic compounds. Meanwhile, compared with the Control and GA_3_ treatment, ABA reduced abundance of five of the seven phenolic compounds catalyzed by those six enzymes (Fig. 1F), suggesting ABA reduced phenolic biosynthesis. In contrast, the phenylpropanoid biosynthesis was one of the KEGG pathways significantly enriched by DEPs, but not by DAMs, in GA_3_ vs Control (Fig. 1D and E). However, compared with the Control, GA_3_ increased abundance of five DEPs for five enzymes (Fig. 1F), and elevated abundance of three phenolic compounds, i. e. p-coumaric acid, p-coumaroyl shikimate and caffeoyl shikimic acid (Fig. 1F), suggesting GA_3_ increased phenolic accumulation. These results were consistent with the evaluation of the TPC showing that ABA reduced and GA_3_ increased TPC in pineapples relative to the control (Fig. S1). Collectively, the results suggest that phenylpropanoid biosynthesis pathway played a key role in differential regulation of pineapple IB by ABA and GA and that phenolic compound accumulation was positively correlated to IB severity.

### Screening for candidate TFs regulating phenolic accumulation in response ABA or GA_3_

**Fig. 2:**
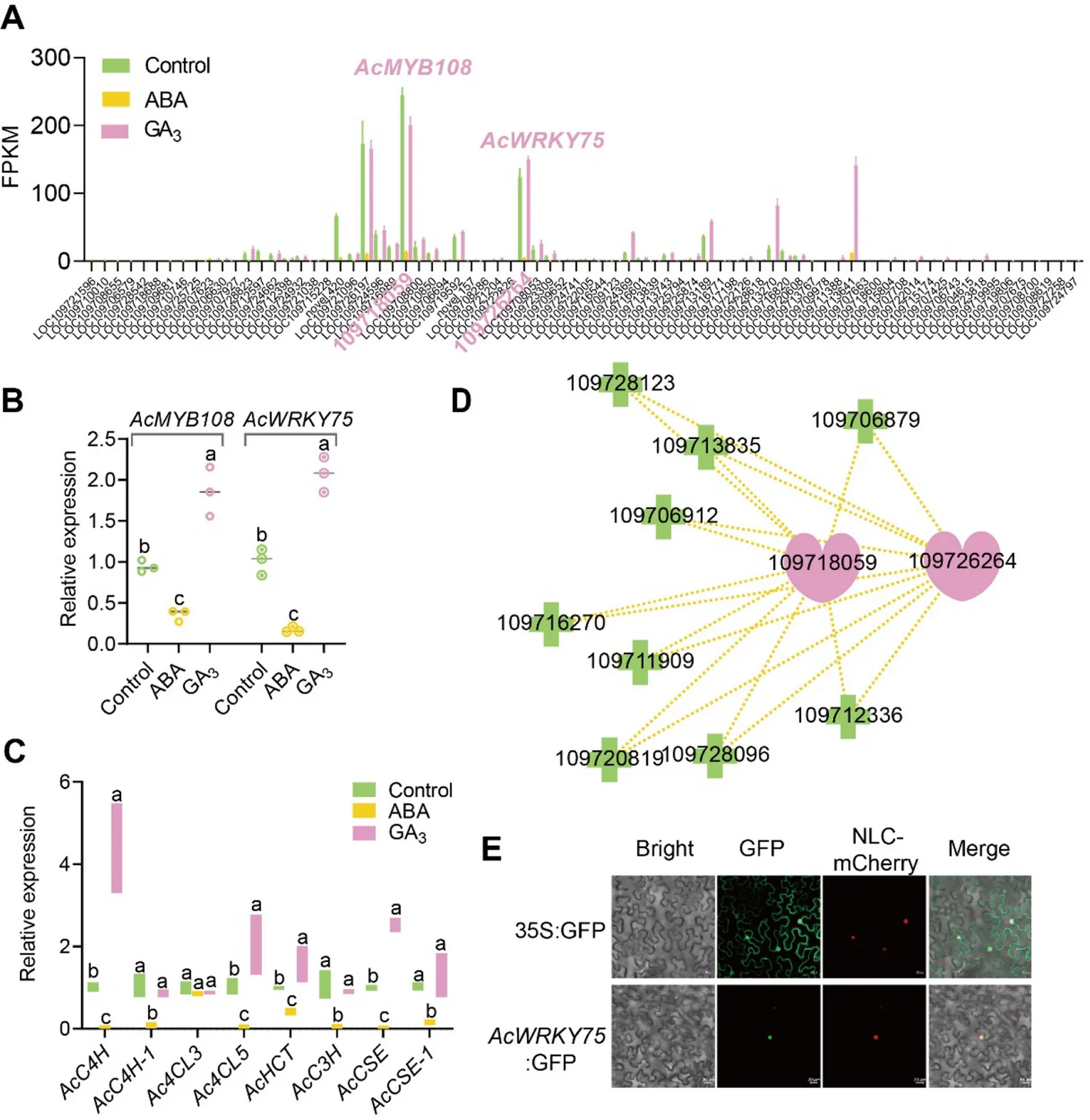
Screening for potential key TFs involved in regulating phenolic accumulation in response ABA or GA_3_. **A** Patterns of expression (FPKM) for different TFs in pineapple. C, Control; A, ABA; G, GA_3_. **B** Expression of *AcMYB108* (LOC109718059) and *AcWRKY75* (LOC109726264) genes. **C** Phenolic biosynthesis related gene expression. *AcC4H* (LOC109706879), *AcC4H-1* (LOC109728096), Cinnamate 4-hydroxylase; *Ac4CL3* (LOC109720819), *Ac4CL5* (LOC109711909), 4-Coumarate CoA ligase; *AcHCT* (LOC109706912), Hydroxycinnamoyl-CoA: shikiate/quinate hydroxycinnamoyl transferase; *AcC3’H* (LOC109728123), 5-O-(4-coumaroyl)-D-quinate 3'-monooxygenase; *AcCSE* (LOC109712336), *AcCSE-1* (LOC109716270), Caffeoyl shikimate esterase. **D** The coexpression network of 2 key TFs (*AcWRKY75* and *AcMYB108*) and 9 potential targeted genes affected by ABA and GA_3_. Light purple heart-shaped circles represent *AcWRKY75* and *AcMYB108*. Green plus sign shape represent 9 potential targeted genes. TF-gene targets showing Pearson correlation > 0.8. **E** Subcellular localization of heterologously expressed *AcWRKY75-GFP* in *N. benthamiana* leaves. mCherry staining was used to indicate the nuclei. Scale bar, 20 μm. Data are given as mean ± SD (n = 3) and significance of differences is indicated by letters above the bars (*P* < 0.05), unless otherwise indicated

Transcriptomic analysis was performed to identify the potential key TFs involved in regulating phenolic accumulation in response ABA or GA_3_. As shown in Fig. 2A, fifty-five differentially expressed TFs (DETF) were identified in ABA *vs* Control, compared sixty-five DETFs in ABA *vs* GA_3_. Although the top 10 DETFs for ABA *vs* Control and ABA *vs* GA_3_ were different, the top three were the same, i. e. *AcWRKY75*, *AcERF114* and *AcMYB108*. We selected *AcWRKY75* and *AcMYB108* TFs for further investigation for the following reasons. First, the transcriptome analysis showed that, compared with the control, GA_3_ downregulated *AcMYB108* and upregulated *AcWRKY75*, but did not obviously change expression of *AcERF114* (Fig. 2A). Secondly, AcERF114 is related to the ethylene signal, while the main focus of this study is on ABA and GA mediation. As for the role that *AcERF114* may play in the process of pineapple IB, another study is ongoing to address it. The RT-PCR analysis showed that *AcWRKY75* and *AcMYB108* were upregulated by GA_3_ and downregulated by ABA in comparison to the Control (Fig. 2B), displaying an expression pattern highly consistent with that of the four key genes related to phenolic biosynthesis, i. e. *AcC4H*, *Ac4CL5*, *AcHCT* and *AcCSE* (Fig. 2C). The co-expression network analysis showed that 9 potential target genes for *AcWRKY75* and *AcMYB108* were related to phenolic biosynthesis (Fig. 2D). The results suggest that *AcWRKY75* and *AcMYB108* may potentially modulate phenolic biosynthesis that are differentially regulated by ABA and GA_3_*.*

### AcWRKY75 increased phenolic accumulation in pineapple via activating *Ac4CL5*

**Fig. 3:**
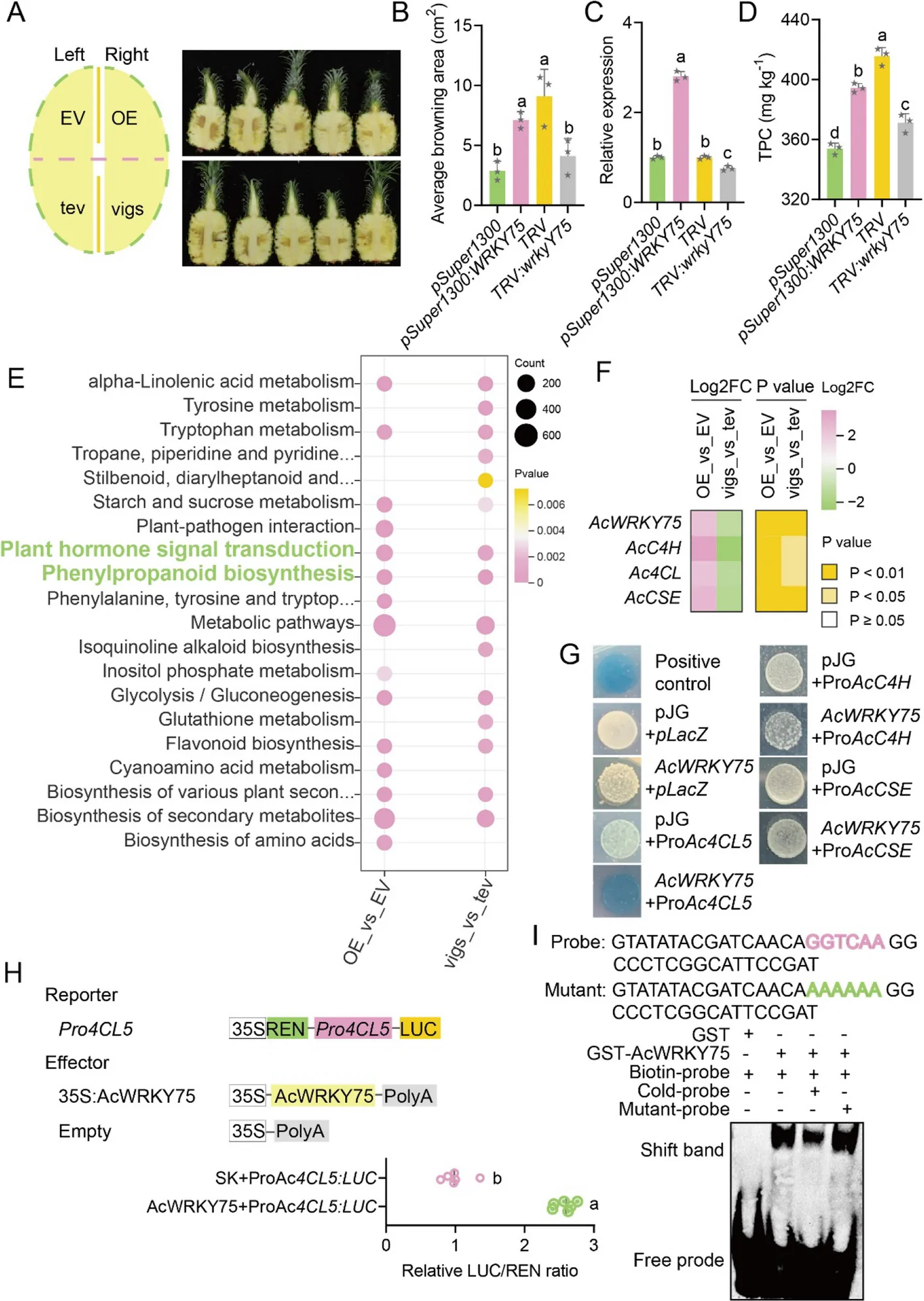
AcWRKY75 positively regulated phenolic accumulation by binding to the W-box mitif in *Ac4CL5* promoter. **A** Phenotypes resulting from transient overexpression and transient silencing of *AcWRKY75* in fruit. The image was taken on 5 d after infiltration with *Agrobacterium tumefaciens* harbouring plasmid *pSuper: AcWRKY75* (right, OE), empty vector (left, EV), *Agrobacterium tumefaciens* harbouring plasmid *pTRV: Acwrky75* (right, vigs) or empty vector (left, tev). The size of the browning area (**B**), the expression of *AcWRKY75* (**C**) and total phenol content (TPC) (**D**) were evaluated at 5 d after transient overexpression and transient silencing of *AcWRKY75*. **E** The top 20 KEGG pathway enrichment of DEGs in pineapple, OE *vs* EV, vigs *vs* tev. **F** The heat map showing the expression of *AcWRKY75*, *AcC4H*, *Ac4CL5* and *AcCSE* genes. **G** Yeast one hybrid (Y1H) assay showing AcWRKY75 binding to the *Ac4CL5* promoter. Both AcWRKY75 + pLacZ and pJG + *Ac4CL5* promoter were used as negative controls. **H** Dual-luciferase activity assay of AcWRKY75 transactivation of the *Ac4CL5* promoter. 35S: AcWRKY75 and Pro*Ac4CL5*: *LUC* were co-infiltrated into *N. benthamiana* leaves. Top, schematic representation of the effector and reporter constructs. Bottom, the dual-luciferase activity (DLA) assay. **I** The electrophoretic mobility shift assay (EMSA) showing the binding of AcWRKY75 to the promoters of *Ac4CL5.* The probe sequences corresponding to promoters of Ac*4CL5* are shown, with red letters representing the W-box sequences, green letters mutant sequences of W-box sites. Symbol ‘–’ represents absence and ‘ + ’ represents presence. Data are given as mean ± SD (n = 3) and significance of differences is indicated by letters above the bars (*P* < 0.05), unless otherwise indicated

We first cloned the ORF sequence of *AcWRKY75* from pineapple fruit. Subcellular localization analysis displayed that the signals of AcWRKY75-GFP were localized to the nucleus of leaf cells of *N. benthamiana*, indicating that AcWRKY75 functions in nucleus (Fig. 2E). To check whether *AcWRKY75* functions in phenolic biosynthesis, we transiently overexpressed and silenced it in pineapple fruit. Compared with EV fruit, the *AcWRKY75* overexpression fruit (OE) displayed bigger browning area (Fig. 3A and B), stronger *AcWRKY75* expression (Fig. 3C), and more TPC (Fig. 3D). On the other hand, the knock-down of *AcWRKY75* in pineapple by *Agrobacterium*-mediated virus-induced gene silencing (VIGS) resulted in smaller browning area (Fig. 3A and B), weaker *AcWRKY75* transcription (Fig. 3C) and less TPC contents (Fig. 3D) than the *pTRV1* + *pTRV2* (tev) fruit. These results suggest that *AcWRKY75* positively regulate phenolic accumulation.

Then we checked the transcriptomic changes in pineapple pulps transiently overexpressing *AcWRKY75* or silencing *Acwrky75.* As shown in Fig. 3E, the biosynthesis of secondary metabolites, metabolic pathways, plant hormone signal transduction and phenylpropanoid biosynthesis were the most enriched four KEGG pathways, for both OE *vs* EV and vigs *vs* tev, confirming that plant hormones were closely related to the role of *AcWRKY75* in regulating phenylpropanoid biosynthesis.

Further analysis showed that *AcWRKY75* and phenolic biosynthetic genes, i. e. *AcC4H*, *Ac4CL5* and *AcCSE*, displayed the same expression patterns, all being up-regulated in OE pineapples and downregulated in vigs fruit (Fig. 3F), suggesting *AcC4H*, *Ac4CL5* and *AcCSE* may be the target genes of *AcWRKY75*. We then explored whether *AcWRKY75* could directly regulate phenolic biosynthesis-related genes with yeast one hybrid (Y1H), dual-LUC (firefly luciferase) reporter (DLR) and electrophoretic mobility shift assay (EMSA). Y1H assay demonstrated that AcWRKY75 directly bound to *Ac4CL5*’s promoter, but not to *AcC3H*’s and *AcCSE*’s (Fig. 3G). DLR assay indicated that AcWRKY75 specifically bound to *Ac4CL5* promoter and activated its transcription (Fig. 3H). Furthermore, the EMSA was conducted to confirm whether AcWRKY75 could specifically bind to W-box motif in vitro. GST- AcWRKY75 fusion protein was generated and purified (Fig. S2). The DNA fragments of *Ac4CL5* promoter covering the W-box motif, the WRKY-recognition sequence (Eulgem et al. 2000), was biotin-labeled as probes. As shown in Fig. 3I, GST- AcWRKY75 fusion protein recognized the W-box motifs in *Ac4CL5* promoter and displayed a mobility shift, while AcWRKY75 binding capacity was reduced after adding cold probes (unlabeled probes), but not affected after adding mutant probes (unlabeled). Furthermore, no mobility shift band was observed for the *Ac4CL5* promoter fragments incubated with GST protein (Fig. 3I). The results strongly indicated that AcWRKY75 specifically bound to promoter of *Ac4CL5* to activate the transcription.

### Overexpression of *AcWRKY75* and *Ac4CL5* in *Arabidopsis* increased phenolic contents

**Fig. 4:**
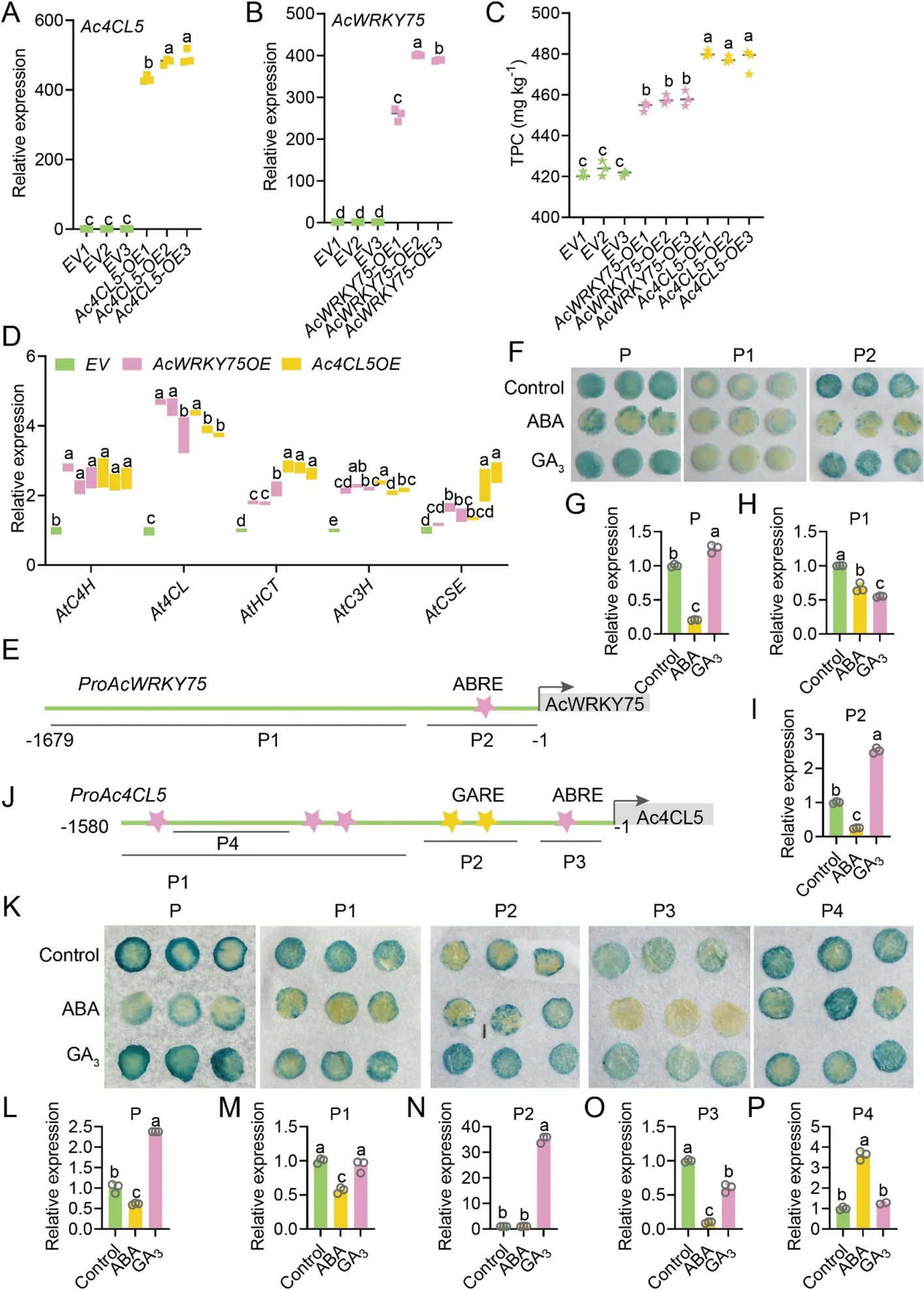
AcWRKY75 and Ac4CL5 positively regulated phenolic accumulation in response ABA or GA_3_. **A-B** Expression of *AcWRKY75* and *Ac4CL5* genes. **C** Effects of *AcWRKY75* and *Ac4CL5* overexpression on total phenol content (TPC) in *Arabidopsis.* **D** Effects of *AcWRKY75* and *Ac4CL5* overexpression on transcription levels of phenolic biosynthetic genes of *Arabidopsis thaliana* seedling. **E** Schematic diagram of the upstream cis elements of *AcWRKY75*. The ABA responsive element (ABRE) motifs were indicated. **F** GUS staining in the tobacco leaves with transient expression of GUS gene driven by Pro*AcWRKY75*, Pro*AcWRKY75*−1 and Pro*AcWRKY75-2*. **G-I** *GUS* expression in *N. benthamiana* leaves. **J** Illustration of promoter segmentation fragment diagram of promoter of *Ac4CL5*. **K** GUS staining in the leaves of tobacco with transient expression of GUS gene driven by Pro*Ac4CL5*, Pro*Ac4CL5*−1, Pro*Ac4CL5*−2, Pro*Ac4CL5*−3 and Pro*Ac4CL5*−4. **L-P** Expression of *GUS* in leaves of *N. benthamiana*. Data are given as mean ± SD (n = 3) and significance of differences is indicated by letters above the bars (*P* < 0.05), unless otherwise indicated

We then constructed the *AcWRKY75*-overexpressing *Arabidopsis* lines (*AcWRKY75-*OE), *Ac4CL5*-overexpressing *Arabidopsis* lines (*Ac4CL5*-OE) and the Control lines (EV: empty vector) (Fig. S3A-D) to verify the function of *AcWRKY75* and *Ac4CL5* (Fig. 4A-D). In OE lines (*AcWRKY75-*OE, *Ac4CL5*-OE) the TPC was significantly increased (Fig. 4C) and the key phenolic synthetic genes*,* i. e. *AtC4H*, *At4CL*, *AtHCT*, *AtC3H* and *AtCSE*, were significantly higher relative to the EV lines (Fig. 4D). The results further confirmed AcWRKY75 and Ac4CL5 positively regulated phenolic biosynthesis in pineapple fruit.

### The transcription of ***AcWRKY75*** and ***Ac4CL5*** were regulated by ABA and GA_3_

As according to sequence analysis, the promoter of *AcWRKY75* contained 1 potential ABRE (ABA responsive element) motifs and that of *Ac4CL5* had 4 potential ABRE motifs and 2 potential GARE (GA responsive element) (Fig. 4E, J), then GUS transient assays were performed in leaves of *N. benthamiana* to check whether ABA or GA_3_ regulates transcription of *AcWRKY75* or *Ac4CL5*.

First, the promoter sequence of *AcWRKY75* was cut into two fragments, with the first containing 0 potential ABRE motifs (Pro*AcWRKY7*5*−1*, P1) and the second one potential ABRE motif (Pro*AcWRKY75*−2, P2) (Fig. 4E). The whole sequence and fragments of *AcWRKY75* promoter were separately fused to GUS reporter gene, i. e. the Pro*AcWRKY75*: GUS, Pro*AcWRKY75-1*: GUS, and Pro*AcWRKY75-2*: GUS, before being introduced into *N. benthamiana* leaves separately. As shown in Fig. 4F-I, ABA- treated tobacco leaves had lower enzyme activity and gene expression of *GUS* driven by Pro*AcWRKY75*, Pro*AcWRKY75-1,* and Pro*AcWRKY75-2* than the Control, indicating that ABA inactivated the transcription of *AcWRKY75*. However, GA_3_- treated *N. benthamiana* leaves had higher enzyme activity and expression of *GUS* driven by Pro*AcWRKY75* and Pro*AcWRKY75-2* relative to the Control (Fig. 4 F, G and I), suggesting that the promoter of *AcWRKY75* may contain potential GA_3_ responsive elements or may be regulated by other factors in response GA.

Then, the promoter sequence of *Ac4CL5* was divided into four fragments, i. e. Pro*Ac4CL5-1* (P1, with 3 potential ABRE motifs), Pro*Ac4CL5*−2 (P2, with 2 potential GARE motifs), Pro*Ac4CL5*−3 (P3, with 1 potential ABRE motifs) and Pro*Ac4CL5*−4 (P4, with no potential ABRE or GARE motifs) (Fig. 4J). The GUS transient assay revealed that ABA-treated tobacco leaves had lower enzyme activity and gene expression of *GUS* driven by Pro*Ac4CL5*, Pro*Ac4CL5-1* and Pro*Ac4CL5-3* relative to the Control, suggesting that ABA inactivated transcription of *Ac4CL5* (Fig. 4K, L, M and O). For GA_3_-treated tobacco leaves, the gene expression of *GUS* driven by Pro*Ac4CL5* and Pro*Ac4CL5-2* were higher than the Control, suggesting that GA_3_ activated the transcription of *Ac4CL5* (Fig. 4K, L and N).

These results together suggest that GA_3_ activated the transcription of *AcWRKY75* and *Ac4CL5* by recognizing the GARE motif, while ABA suppressed the transcription of *AcWRKY75* and *Ac4CL5* by recognizing the ABRE motif.

### Overexpression of *AcMYB108* increased phenolic accumulation in *Arabidopsis*

**Fig. 5:**
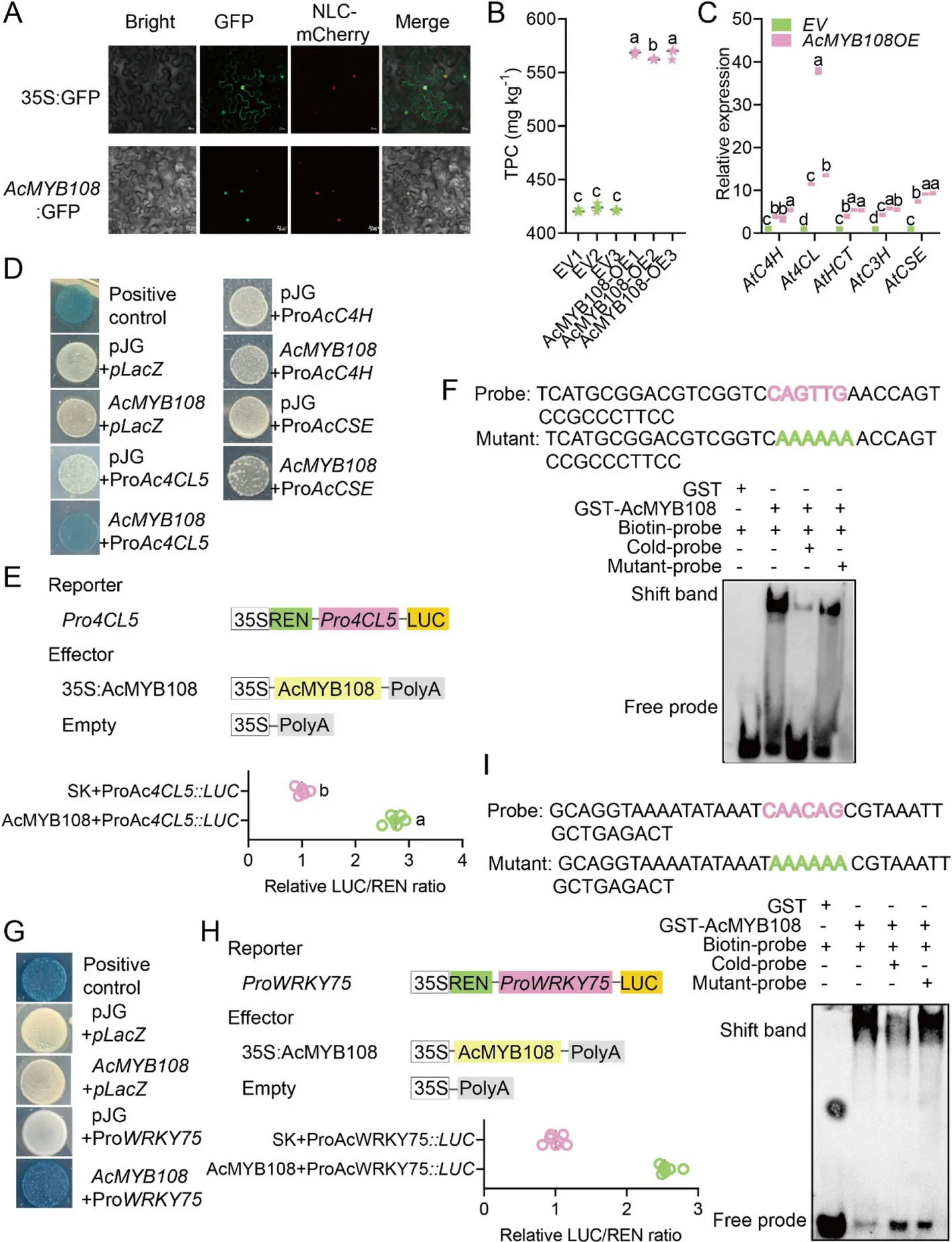
AcMYB108 positively regulated phenolic accumulation by binding to the MYB binding motif in *Ac4CL5* promoter or *AcWRKY75* promoter. **A** Subcellular localization of heterologously expressed *AcMYB108-GFP* in tobacco leaves. mCherry staining was used to display the cell nuclei. Scale bar, 20 μm. **B** Effects of *AcMYB108* overexpression on total phenol content (TPC) in *Arabidopsis.* **C** Effects of *AcMYB108* overexpression on transcription level of phenolic biosynthesis genes of *Arabidopsis thaliana*. **D** The Y1H assay showing AcMYB108 binding to the Ac*4CL5* promoter. Both AcMYB108 + pLacZ and pJG + Ac*4CL5* promoter were used as negative controls. **E** The DLR assay of AcMYB108 activation of the *Ac4CL5* promoter. 35S: AcMYB108 and ProAc*4CL5*: *LUC* were co-infiltrated into *N. benthamiana* leaves. Top, schematic representation of the effector and reporter constructs, Bottom, the dual-luciferase activity assay (right). **F** The EMSA showing the binding of AcMYB108 to the promoters of Ac*4CL5.* The probe sequences corresponding to promoters of Ac*4CL5* are shown, with red letters representing the MYB-binding sequences and green letters mutant sequences of MYB-binding sites. Symbol ‘–’ and ‘ + ’ represent absence or presence, respectively. **G** The Y1H assay showing AcMYB108 bound to promoter of *AcWRKY75*, with both AcMYB108 + pLacZ and pJG + Ac*WRKY75* promoter as negative controls. **H** The DLA assay of AcMYB108 activation of promoter of *AcWRKY75*. Pro*AcWRKY75*: *LUC* and 35S: AcMYB108 were co-transformed into leaves of *N.benthamiana*. Top, schematic illustration of the constructs of effector and reporter, Bottom, the results of DLA assay. **I** The EMSA showing AcMYB108 bound to *AcWRKY75* promoter*.* The probe sequences corresponding to promoters of *AcWRKY75* are shown, where the red letters represent the MYB-binding sequences and green letters mutant sequences of MYB-binding sites. The symbol ‘–’ represents absence and ‘ + ’ presence. Data are given as mean ± SD (n = 3) and significance of differences is indicated by letters above the bars (*P* < 0.05), unless otherwise indicated

Then we cloned the ORF sequence of *AcMYB108* from pineapple fruit. As shown in Fig. 5A, AcMYB108 protein was localized to the nucleus, whereas the empty GFP was localized in the entire cell, indicating that AcMYB108 functions in nucleus. To investigate the roles of AcMYB108 TF in biosynthesis of phenolic compounds, we generated transgenic *Arabidopsis* lines overexpressing *AcMYB108* (Fig. S4A to C, S3B). Results showed that the TPC and expression of phenolic biosynthesis-related genes (*AtC4H*, *At4CL*, *AtHCT*, *AtC3H* and *AtCSE*) were tremendously higher than the EV lines (Fig. 5B, C). These results confirmed that AcMYB108 positively regulated phenolic accumulation in pineapple fruit.

### AcMYB108 directly regulated expression of *Ac4CL5*

To further confirm the role of AcMYB108 in regulating phenolic biosynthesis, the mode of action of *AcMYB108* TF was investigated. *AcC4H*, *Ac4CL5* and *AcCSE* may be the target genes of *AcMYB108*, as they showed expression patterns similar to that of *AcMYB108* (Fig. 2C, D). The Y1H assay demonstrated that *AcMYB108* directly bound to the promoter of *Ac4CL5*, but not to that of *AcC3H* and of *AcCSE* (Fig. 5D). The binding activity was further confirmed both by the DLR assay (Fig. 5E) and the EMSA assay (Fig. 5F), indicating that *AcMYB108* activated *Ac4CL5* transcription by recognizing the MYB binding motifs (CAACTG) in *Ac4CL5* promoter (Fig. S5,5F). These results suggest that AcMYB108 functioned as an activator for phenolic biosynthesis in pineapple fruit.

### AcMYB108 directly regulated expression of *AcWRKY75*

To check the relationship between *AcMYB108* and *AcWRKY75*, the assays of Y1H, DLR and EMSA were performed. The results of Y1H assay showed AcMYB108 directly bound to the promoter of *AcWRKY75* (Fig. 5G). As shown in Fig. 5H, co-transformation of *N. benthamiana* leaves with the 35S: *AcMYB108* effector and the Pro*AcWRKY75*: LUC reporter produced significantly increased luciferase activity relative to the Control (empty SK effector + Pro*AcWRKY75*: LUC reporter), suggesting *AcMYB108* specifically activated *AcWRKY75*. Moreover, the EMSA results in Fig. 5I showed that migration of the protein–DNA complexes was reduced when the AcMYB108 fusion protein (Fig. S5) was mixed with biotin-labeled probes including the MYB-binding motif (CAACAG) derived from the *AcWRKY75* promoter, but not affected when adding mutant probes (unlabeled). These strongly suggest that *AcMYB108* directly bound to *AcWRKY75* promoter and activated its transcription.

### The transcription of *AcMYB108* was regulated by GA_3_ and ABA

**Fig. 6:**
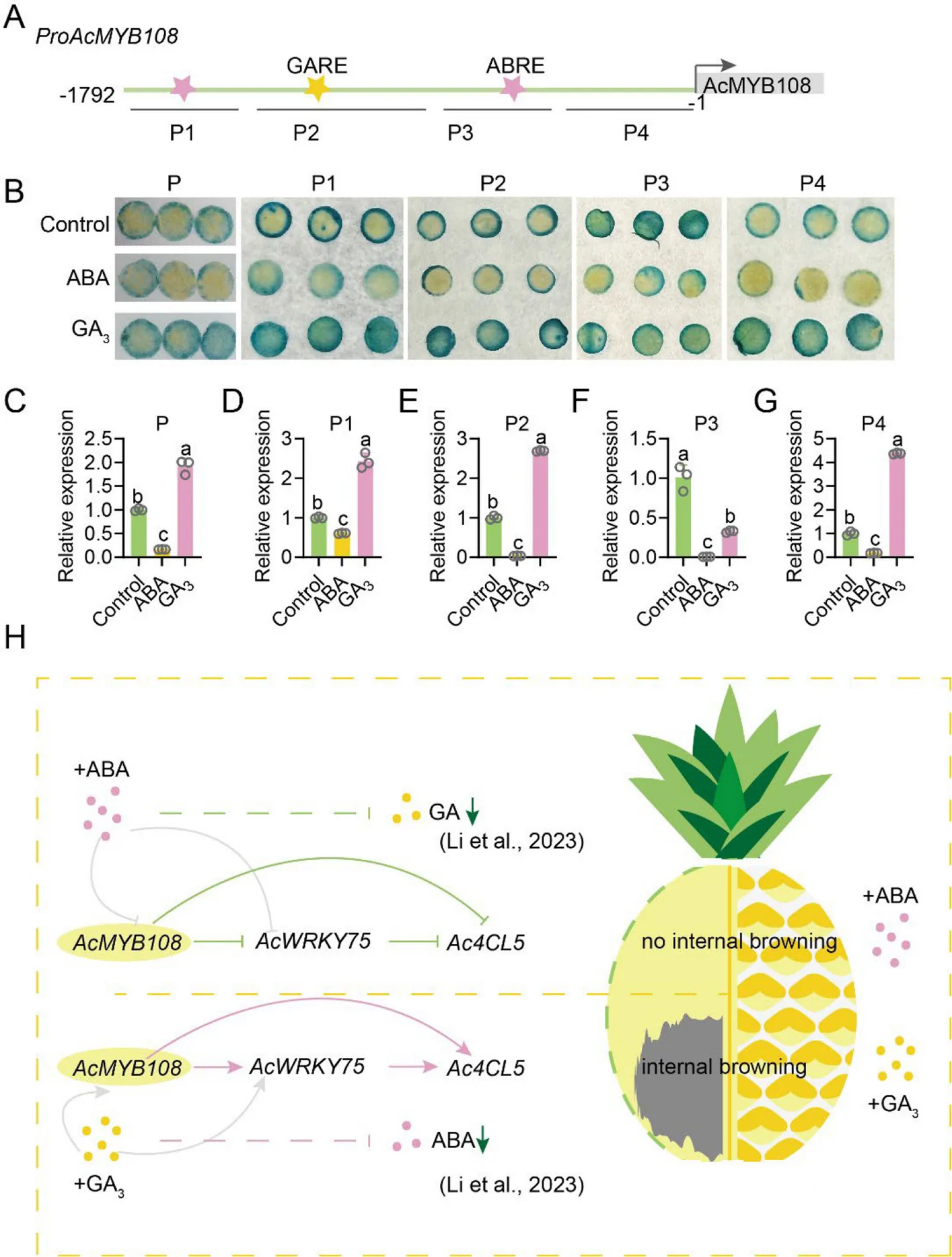
AcMYB108 regulated *Ac4CL5* and *AcWRKY75* in response ABA and GA_3_. **A** Illustration of promoter segmentation fragment diagram of promoter of *AcMYB108*. **B** GUS staining in the leaves of tobacco with transient expression of GUS gene driven by Pro*AcMYB108,* Pro*AcMYB108-1*, Pro*AcMYB108-2*, Pro*AcMYB108-3* and Pro*AcMYB108-4*. **C-G** The expression of *GUS* in leaves of *N.benthamiana*. **H** A proposed model for pineapple internal browning antagonistically regulated by ABA and GA_3_. Data are given as mean ± SD (n = 3) and significance of differences is indicated by letters above the bars (P < 0.05), unless otherwise indicated

As aforementioned, the *AcMYB108* transcription was induced by GA_3_ and suppressed by ABA (Fig. 2B), but how ABA or GA_3_ regulated *AcMYB108* expression remains unclear. We used the PlantCARE database to analyze the *AcMYB108* promoter sequence, which revealed 2 potential ABRE motifs and 1 potential GARE (Fig. 6A). The GUS assay was performed to verify the regulation of *AcMYB108* by ABA or GA_3_. First, the promoter sequence of *AcMYB108* was divided into four fragments, i. e. ProAc*MYB108-1* (P1, with 1 potential ABRE motif), ProAc*MYB108*−2 (P2, with 1 potential GARE motifs), Pro*AcMYB108*−3 (P3, with 1 potential ABRE motifs) and ProAc*MYB108*−4 (P4, with no potential ABA or GA motifs) (Fig. 6A). The ABA-treated tobacco leaves had lower enzyme activity and gene expression of *GUS* driven by Pro*AcMYB108*, Pro*AcMYB108-1,* Pro*AcMYB108-2,* Pro*AcMYB108-3* and Pro*AcMYB108-4* relative to the Control, suggesting that ABA inactivated the transcription of *AcMYB108* (Fig. 6B-G). On the other hand, the GA_3_-treated tobacco leaves showed higher expression of *GUS* driven by Pro*AcMYB108*, Pro*AcMYB108-2* and Pro*AcMYB108-4* than Control (Fig. 6B, E and G), suggesting that GA_3_ activated the transcription of *AcMYB108*. These results together suggest GA_3_ and ABA oppositely modulated phenolic synthesis via regulating *AcMYB108* transcription. In addition, the results imply that Pro*AcMYB108-4* might contain potential ABA motifs and that Pro*AcMYB108*, Pro*AcMYB108-2* and Pro*AcMYB108-4* might contain potential ABA motifs.

## Discussion

It has been well documented that ABA and GAs are antagonistic in regulating a wide range of biological processes. We showed previously that ABA alleviated and GA_3_ aggravated pineapple IB via differentially regulating spermidine accumulation (Li et al. 2023b). In this study, we further showed by proteomic and metabolomic analysis that ABA and GA_3_ differentially modulated pineapple IB through regulating phenylpropanoid biosynthesis pathway. First, as ABA reduced IB relative to the Control and GA_3_, analyses of the proteomic and metabolomic data between ABA vs Control and ABA vs GA_3_ would help identify the pathways related to suppression of IB by ABA. The comparison between the 16 KEGG pathways significantly enriched by DEPs displayed three shared pathways, i. e. phenylpropanoid biosynthesis, ascorbate and aldarate metabolism, and ribosome (Fig. 1D), of which phenylpropanoid biosynthesis was the only shared KEGG pathway significantly enriched by DAMs (Fig. 1E). This suggests a critical role for phenylpropanoid biosynthesis in suppression of IB by ABA and that the proteins or enzymes in ascorbate and aldarate metabolism, and ribosome pathways were involved in that process. Secondly, since accumulation of phenolic compounds are closely related to plant tissue browning (Fang et al. 2022), and they form one of the major categories of secondary metabolites produced in phenylpropanoid pathway, it is necessary to identify specific proteins and phenolic metabolites related to ABA treatment. 13 proteins for six key enzymes related to phenolic biosynthesis were identified in ABA-treated fruit and all but one was downregulated, which was in line with the reduced abundance of five of the seven phenolic compounds, suggesting ABA inactivated phenylpropanoid biosynthesis pathway (Fig. 1F). Thirdly, since GA_3_ increased the IB severity relative to the Control, it is of interest to check what pathways were involved at both proteomic and metabolomic levels. The phenylpropanoid biosynthesis was one of the KEGG pathways significantly enriched by DEPs, but not by DAMs in GA_3_-treated pineapples vs the Control (Fig. 1D, E). However, GA_3_ upregulated five DEPs (Fig. 1F) and increased abundance of three phenolic compounds, suggesting GA_3_ activated phenolic biosynthesis. Fourthly, that ABA and GA_3_ regulated phenolic biosynthesis in opposite direction was confirmed by the assay of TPC in ABA- and GA-treated pineapples (Fig. S1). Therefore, it can be proposed that the differential regulation of pineapple IB by GA_3_ and ABA was mainly due to the opposite modulation of phenolic biosynthesis. The findings showed that pineapple IB regulated by ABA and GA involves differential modulation of phenylpropanoid biosynthesis pathway.

To investigate how ABA and GA regulated phenolic biosynthesis, we first checked which TFs were most significantly influenced by ABA and GA in pineapples. Of the 55 and 65 DETF identified from ABA vs Control and ABA vs GA_3_ (Fig. 2A), the top three, i.e. *AcWRKY75*, *AcERF114* and *AcMYB108,* may potentially play roles in regulating phenolic biosynthesis (Table S2, S3). Based on the expression patterns of TFs and the aim of this study, we addressed the role of *AcWRKY75* and *AcMYB108* in regulating phenolic biosynthesis. *AcWRKY75* transient overexpression enhanced IB and increased TPC in pineapple (Fig. 3A-D), while its transient silencing alleviated IB (Fig. 3A, B) and reduced TPC (Fig. 3D). Ectopic expression of *AcWRKY75* and *AcMYB108* in *Arabidopsis* increased TPC (Figs. 4C, and 5B) and enhanced expression of phenolic biosynthetic genes (Figs. 4D and 5C). The assay of the Y1H, DLR and EMSA all consistently showed that both AcWRKY75 and AcMYB108 activated *Ac4CL5* transcription (Figs. 3G-I and 5D-F). The *Ac4CL5* OE lines displayed more TPC (Fig. 4C) and higher expression of *AtC4H*, *At4CL*, *AtHCT*, *AtC3H* and *AtCSE* (Fig. 4D). Previous studies showed that overexpression of Pb4CL11 in *A. thaliana* increased the expression of *AtPAL*, *AtC4H*, *AtHCT*, *AtC3H*, *AtCCOMT*, *AtCCR*, *AtF5H*, *AtCOMT*, *AtCAD4* and *AtCAD5* and promoted lignin biosynthesis (Cao et al. 2023). Overexpression of Ok4CL11 in *N. benthamiana* resulted in upregulation of *PAL2*, *C4H*, *4CL*, *CHS1*, *CHS2*, *CHI2*, *GT3*, *GT6*, *GT7* genes, and that of Ok4CL7 down-regulated *PAL1*, *PAL2*, *CHI2*, *F30H*, *FLS1*, and *GT7* genes (Lavhale et al. 2024). How does 4CL as an enzyme in phenylpropanoid biosynthesis pathway affect expression of other genes remains to be further elucidated. These results strongly suggest AcWRKY75 and AcMYB108 as activators for phenolic biosynthesis in pineapple fruit. ABA promotes the *FaMYB10* expression, leading to accumulation of anthocyanin via enhancing gene expression in flavonoid pathway (Kadomura-Ishikawa et al. 2015). GA_3_ promotes lignin accumulation by inducing *JrMYB180* expression (Li et al. 2023a). How ABA and GA regulate phenolic biosynthesis via MYB and WRKY TFs remains largely unknown. Therefore, we investigated how ABA and GA regulated AcWRKY75 and AcMYB108. The sequence analysis revealed that the *AcWRKY75* promoter contained 1 potential ABRE motifs (Fig. 4E), and the *AcMYB108* promoter 2 potential ABRE motifs and 1 potential GARE (Fig. 6A). The transient expression assay of GUS driven by promoters showed that GA_3_ activated and ABA suppressed the transcription of *AcWRKY75* (Fig. 4E-I), *Ac4CL5* (Fig. 4J-P) and *AcMYB108* (Fig. 6A-G). The results suggest that GA_3_ and ABA oppositely modulated phenolic biosynthesis. It is interesting that, the *Ac4CL5* promoter contained 4 potential ABRE motifs and 2 potential GARE, and that GA_3_ activated and ABA suppressed the transcription GUS driven by its promoter transiently expressed in tobacco (Fig. 4J -P), suggesting ABA and GA can directly regulate phenolic biosynthesis, also in opposite direction. It was previously shown in pineapple fruit as well as in potato (*Solanum tuberosum* L.) tubers that exogenous ABA increased endogenous ABA contents, while exogenous GA_3_ reduced endogenous ABA contents and enhanced biosynthesis of endogenous bioactive GAs in pineapple (Che et al. 2022; Li et al. 2023b), suggesting ABA and GA antagonize each other in biosynthesis of themselves in pineapple fruit at senescent stage. Therefore, it can be suggested that ABA and GA_3_ antagonistically modulated phenolic biosynthesis to regulate IB through opposite regulation of the two TFs AcWRKY75 and AcMYB108 and the target gene *Ac4CL5*.

Previous studies showed that members of WRKY or MYB TF families can target two related genes in phenylpropanoid biosynthesis pathway. For example, GhWRKY41 activates the transcription of *Gh4CL* and *GhC4H* to regulate lignin and flavonoid accumulation (Xiao et al. 2023), and PpMYB308 promotes phenolic accumulation by directly binding to *PpPAL1 and Pp4CL5* promoters (Li et al. 2023d)*.* In this study, both AcWRKY75 and AcMYB108 activated the same target *Ac4CL5* (Figs. 3G-I and 5D-F). Previously, WRKY and MYB TFs have been shown to separately regulate biosynthesis of secondary metabolites. For instance, Medicago *WRKY* positively regulates caffeic acid accumulation in transgenic tobacco (Naoumkina et al. 2008), and FaMYB10 promotes anthocyanin accumulation of *Fragaria x ananassa* fruit (Kadomura-Ishikawa et al. 2015). In this study, we showed that AcMYB108 directly bound to *AcWRKY75* promoter to activate its transcription (Fig. 5G-I). These results showed that AcWRKY75 and AcMYB108 not only activated phenolic biosynthesis independently, but also synergistically, suggesting coordinated transcriptional activation for phenolic biosynthesis. We showed previously that *AcWRKY75*, *AcMYB108* and *Ac4CL5* were upregulated and the phenolic biosynthesis pathway activated in pineapples with IB (Li et al. 2023c). Together, these suggest that the high susceptibility of harvested pineapple to IB was closely related to the two activators AcWRKY75 and AcMYB108 that worked together to strengthen activation of phenolic biosynthesis. It is noted that phenolic compounds were accumulated not only in senescent pineapple, but also in other senescent fruits, such as apple (Fang et al. 2022) and peach (Liu et al. 2015). Since phenolic compounds are secondary metabolites that play important roles in plant’s defense against pathogenic diseases (Baidez et al. 2007) and insects (Hammerbacher et al. 2013), the increase in phenolics in senescent stage of fruit might have evolutionary significance, as it may render protection to reproductive organs. However, the evolutionary implication of the phenolic biosynthesis inhibited by ABA and enhanced by GA in senescent fruit remains to be further elucidated.

Taken together, the results of this work suggest a novel model for transcriptional regulation of phenolic biosynthesis, in which there exist multiple layers of modulation, which involves the biosynthetic gene *Ac4CL5*, the TFs *AcWRKY75* and *AcMYB108* and the phytohormones ABA and GA (Fig. 6). In this model, *Ac4CL5*, the key phenolic biosynthetic gene, was regulated not only by AcWRKY75 and AcMYB108, but also oppositely modulated by ABA and GA. In addition, AcWRKY75 and AcMYB108 were also oppositely regulated by ABA and GA. Moreover, *AcWRKY75* was activated by AcMYB108. This model showed that phenolic biosynthesis was double guaranteed, implying phenolic biosynthesis as an important biological process in pineapple fruit during postharvest processes. These findings provide novel insight into mechanism underlying modulation of pineapple IB.

## Methods

### Plant material

Fruit of pineapple (*Ananas comosus* L.) at commercial maturity (70% maturity) were harvested and transported to Postharvest Pathology and Molecular Biology Laboratory within 12 h of harvest. Fruit uniform in size and free of disease were selected as experimental material, rinsed with tap water and air-dried before being used for further treatment. (A) For conducting ABA and GA_3_ treatment, pineapples were sprayed with a fine mist of ABA solution (0.2 g L^−1^), GA_3_ solution (0.2 g L^−1^) or distilled water (Control) until runoff and air-dried. Samples of pulps were collected every 3 d. (B) For performing transient expression test, one side of pineapple was injected at the equatorial line with empty vector (*pSuper1300* or *pTRV*), and the other side was injected with *pSuper1300:AcWRKY75*, *pTRV:Acwrky75* or *pSuper1300:Ac4CL5*. There were three replications for each treatment that contained 15 fruit. Samples of pulps were collected daily.

Tobacco (*Nicotiana benthamiana*) and *Arabidopsis* plants were grown under white fluorescent light illumination with 16 h light and 8 h of darkness. Samples were frozen in liquid nitrogen and stored at − 80 °C.

### Assessment of IB incidence and index

IB incidence and index were calculated as described previously (Li et al. 2023c). IB incidence refers to the percentage of internal browning fruit. The IB index represents IB intensity, which was visually scored from 0 (no internal browning) to 5 (maximum internal browning) of each fruit as described by Teisson (1979).

### TPC assay

The TPC (total phenol content) was determined following the protocol described previously (Li et al. 2023c). In brief, the sample (0.5 g) of pineapple pulp was incubated in 2.5 mL ethanol solution (60%) at 60◦C for 2 h and centrifuged at 13000 rpm for 20 min. Then, the mix solution (0.1 mL supernatant 0.1 mL; 0.9 mL double-distilled water, 1 mL (0.2 M) Folin–phenolreagent; 0.7 mL (1 M) sodium carbonate solution) was incubated for 30 min (25^◦^C). Then, the absorbance at 765 nm was measured and TPC was expressed as milligrams of gallic acid equivalent per gram of fresh sample.

### Metabolomics analysis

Metabolome analysis of pineapple fruit was performed as previously described (Li et al. 2023b). VIP (variable importance in projection) ≥ 1 and absolute log2 fold change ≥ 1 was set as the threshold for significantly accumulated metabolites.

### Transcriptomic analysis

The tissues of pineapple pulp treated with distilled water (Control), ABA (0.2 g L^−1^) and GA (0.2 g L^−1^) at 9 days of storage were sampled for transcriptomic analysis. Three replicates from each treatment were separately sampled. Transcriptomic analysis of pineapple fruit was performed as we described previously (Li et al. 2023b). | Log2 (Fold Change) |> 0 and *Padj* < 0.05 were used as a screening criterion for differentially expressed genes. Notably, | Log2 (Fold Change) |> 3.5 and *Padj* < 0.05 were used as a screening criterion for differentially expressed TFs in Fig. 2A and B.

### Proteomic analysis

The tissues of pineapple at 9 days of storage were sampled for proteomic analysis. Three replicates from each treatment were separately sampled. Protein extraction, digestion and TMT labeling was essentially conducted as previously described (Guo et al. 2023). Protein sequences were retrieved from *Ananas*_comosus_4615_PR_20210121.fasta. P-value ≤ 0.05 and absolute Log2FC (fold change) > 1.3 was used as a screening criterion for significantly accumulated proteins. Notably, | Log2 (Fold Chang) |> 0.3 and *Padj* < 0.05 were used as a screening criterion for DEPs in Fig. 1F.

### RT-qPCR analysis

The expression of genes was assessed through real-time quantitative PCR (RT-qPCR) (Li et al. 2023c). Three replicates of each sample were analyzed. Relative transcript levels of genes were calculated with 2^−ΔΔCt^ methods. Primer pairs were shown in Table S1.

### Subcellular localization of AcWRKY75 and AcMYB108

Full-length open reading frame (ORF) of *AcWRKY75* and *AcMYB108* were cloned into the Super1300 vector (including green fluorescent protein, GFP) to obtain the pSuper: *AcWRKY75-GFP* and pSuper: *AcMYB108-GFP* plasmid, respectively. Subcellular localization of AcWRKY75-GFP and AcMYB108-GFP were determined as described previously (Li et al. 2023c). *Agrobacterium* cells (GV3101), which contained pSuper: AcWRKY75*-GFP* or pSuper: AcMYB108*-GFP* recombinant plasmid, were fused in solution (200 μM acetosyringone, 10 mM MES-KOH and 10 mM MgCl_2_, pH 5.6) to an OD_600_ = 0.8, before being penetrated into *N. benthamiana* leaves. 48 h after agroinfiltration, the fluorescence signals (GFP: 488 nm, DAPI: 408 nm) were captured through confocal laser-scanning microscope (Nikon, Tokyo, Japan). Primer pairs were shown in Table S1.

### Transient transformation analysis in pineapple fruit

The core coding sequence (CDS) of *AcWRKY75* (*LOC109726264*) was cloned using the cDNAs from pineapple fruit and sequenced by Sangon Biotech (Shanghai) Co. Ltd. A 630 bp fragment of the *AcWRKY75* CDS was inserted into pSuper 1300 vector. The resulting constructs were transformed in *Agrobacterium tumefaciens* (strain GV3101). *Agrobacterium* cells (GV3101), which contained pSuper: AcWRKY75*-GFP* recombinant plasmid, were fused in solution (200 μM acetosyringone, 10 mM MES-KOH and 10 mM MgCl_2_, pH 5.6) to an OD_600_ = 0.8, before being penetrated into pineapple. The suspension was then infiltrated into the pineapple with a 1 ml syringe using gentle pressure to make the suspension expand into the pineapple flesh as much as possible. Two pinholes were made and infiltrated on each fruit. The side of fruit infiltrated with empty pSuper 1300 vector was used as negative control. Samples of pulps were collected daily. An *AcWRKY75* fragment (1–300 bp) was amplified and fused in pTRV2 vector to construct the pTRV2:*Acwrky75* plasmid. VIGS assay of *Acwrky75* in pineapple fruit was conducted following the protocol described previously (Bai et al. 2019). The combination (Empty pTRV1 and pTRV2) served as a negative control (tev). Primer pairs were shown in Table S1.

### Transcriptomic analysis of pineapple tissues transiently expressing WRKY75

The tissues of pineapple pulp at 5 days after transiently expressing WRKY75 were sampled for transcriptomics analysis. Transcriptomic analysis of pineapple fruit tissues was performed as described in 2.5. | Log2 (Fold Chang) |≥ 1 and *FDR* < 0.05 were used as a screening criterion for differentially expressed genes.

### Browning area of pineapple fruit transiently expressing AcWRKY75

For evaluating browning area of pineapple resulted from transient expression of *AcWRKY75*, the pineapple was cut lengthwise along the injection hole, the length and width of the browning parts were determined to calculate the average browning area of the cut surface.

### Y1H assay

Full-length ORF of *AcWRKY75* was fused to pJG4-5 vector (with *Xho* I and *Eco*R I sites) (Clontech Laboratories, Inc. Mountain View, CA, USA) to obtain GAD- *AcWRKY75* construct. Full-length ORF of *AcMYB108* was fused to pJG4-5 vector (containing *Xho* I and *Eco*R I sites) to obtain GAD- *AcMYB108* construct. The promoters of *AcC4H, Ac4CL5, AcCSE* and *AcWRKY75* were ligated into pLacZi2µ vector, respectively. Y1H assays were performed following the methods as previously described (Li et al. 2023c). The combination (*RhHB1* + *RhGA20ox1* promoter) served as a positive control. Primer pairs for Y1H were shown in Table S1.

### DLR assay

*AcWRKY75* or *AcMYB108* ORF was cloned into pGreen II 62-SK vector to generate the effector, and the promoter fragments of *Ac4CL5* or *AcWRKY75* were subcloned into pGreen II 0800-LUC vector to obtain the reporter, respectively. *Agrobacterium* GV3101 (pSoup) including effector and reporter was evenly mixed in a volume ratio of 8:2, and the solution was co-transformed into leaves of tobacco. The combination (pGreen II 62-SK + ProAc4CL5: LUC; pGreen II 62-SK + ProAcWRKY75: LUC) was used as the control. 72 h hours after agroinfiltration, LUC was determined through the Dual Luciferase Reporter Gene Assay Kit (Yeasen, Shanghai, China). Primer sequences for DLR were shown in Table S1.

### EMSA

*AcWRKY75* or *AcMYB108* ORF were fused to pGEX-4 T-1 empty vector to establish the GST- AcWRKY75 or GST- AcMYB108 plasmid, which was then transferred to cells (DE3: *Escherichia coli* strain Rosetta). With Isopropyl-β-D-thiogalactopyramoside (0.5 M) as an inducer, and the cells were cultured at 16 ℃. Then, recombination proteins were purified through the GST 4FF Sefinose (TM) Resin Kit (Sangon Biotech, Shanghai, China). The probes harboring the putative WRKY binding site W-box (TTGACC) or MYB binding site (CAACTG) originated from the *Ac4CL5* promoter sequence were biotin labeled through EMSA Probe Biotin Labeling Kit (Beyotime, Shanghai, China). The probes harboring the putative MYB binding site (CAACAG) originated from the *AcWRKY75* promoter sequence were biotin labeled through EMSA Probe Biotin Labeling Kit. DNA-binding assay was conducted through Chemiluminescent EMSA Kit (Beyotime) according to the protocols. Primer pairs were shown in Table S1.

### Detection of ProAcWRKY75, ProAcMYB108 and ProAc4CL5 responsiveness to ABA and GA_3_

The *AcWRKY75*, *AcMYB108* and *Ac4CL5* promoter fragments were fused to pCAMBIA1391 vector containing the reporter gene (β-glucuronidase, GUS), respectively. GUS transient expression test was performed as previously described (Li et al. 2023c). Distilled water (control), ABA (0.2 g L^−1^) or GA_3_ (0.2 g L^−1^) solution was evenly sprayed on the leaves of tobacco 6 h after injection. 48 h after treatment, the leaves of tobacco were stained by GUS Staining Kit (Ready-To-Use). The total RNA of tobacco leaves was extracted, and the GUS reporter gene expression was detected through RT-qPCR as described in Section “AcMYB108 directly regulated expression of Ac4CL5*”*. The primers are in Table S1.

### Generation of transgenic *Arabidopsis*

Full-length CDS of *AcWRKY75, AcMYB108* and *Ac4CL5* was inserted into pSuper 1300 vector, respectively, before the latter being introduced into the cells (GV3101: *A. tumefaciens* strain). Positive transgenic *Arabidopsis* lines overexpressing *AcWRKY75, AcMYB108* and *Ac4CL5* were generated following the methods described previously (Li et al. 2023c). RT-qPCR was carried out to determine the expression of genes (*AcWRKY75, AcMYB108*, *Ac4CL5, AtC4H*, *At4CL*, *AtHCT*, *AtC3H*, *AtCSE*) in transgenic lines. Primer pairs were shown in Table S1.

### Statistical analyses

Data were analyzed through the software of GraphPad Prism (GraphPad Software, Inc. La Jolla, CA, USA). The mean ± standard deviation (SD) was used to represent the result. Data were analyzed through one-way analysis of variance (ANOVA). DPS software (version 9.01) was applied to statistical analysis (P < 0.05) of Duncan's multiple range tests.

## Supplementary Information

Supplementary Material 1: Fig. S1 The TPC in pineapples as affected by ABA and GA_3_ treatment. Fig. S2 Purification of the recombinant GST-AcWRKY75 protein used for the EMSA assay. Fig. S3 AcWRKY75 and Ac4CL5 positively regulated phenolic accumulation in Arabidopsis. Fig. S4 AcMYB108 positively regulated phenolic accumulation in Arabidopsis. Fig. S5 Purification of the recombinant GST-AcMYB108 protein used for the EMSA assay. Table S1 Sequences of primers used this study. Table S2 The top 10 differentially expressed TFs identified from ABA treatment vs Control. Table S3 The top 10 differentially expressed TFs identified from ABA treatment vs GA_3_.

## Data Availability

The data will be available from the corresponding author upon reasonable request.

## Abbreviations

GA: Gibberellic acid
ABA: Abscisic acid
IB: Internal browning
PAL: Phenylalanine ammonia lyase
C4H: P-coumaroyl CoA by cinnamate 4-hydroxylase
4CL: 4-Coumarate: coenzyme A (CoA) ligase
TFs: Transcription factors
DEPs: Differentially expressed proteins
DAMs: Differentially accumulated metabolites
TPC: Total phenol content
DETF: Differentially expressed TFs
OE: Overexpression fruit
VIGS: Virus-induced gene silencing
YIH: Yeast one hybird
DLR: Dual-LUC (firefly luciferase) reporter
EMSA: Electrophoretic mobility shift assay
ORF: Full-length open reading frame
CDS: Core coding sequence CDS
