# Supplementary material for: AcWRKY75 and AcMYB108 mediated ABA-GA antagonism in the regulation of pineapple internal browning
Source: Mol Hortic. 2026 Oct 11;6:73. doi: 10.1186/s43897-026-00247-6 (PMC13654230; doi:10.1186/s43897-026-00247-6)
Supplement: Supplementary file 1 [file 43897_2026_247_MOESM1_ESM.docx]

**Supplementary materials**

**Fig. S1** The total phenolic contents (TPC) in pineapples as affected by ABA and GA_3_ treatment. Data are given as mean ± SD (n=3). Significance of differences is indicated by letters above the bars (*P*<0.05).

**Fig. S2** Purification of the recombinant GST-AcWRKY75 protein used for the EMSA assay. Marker: protein marker; 1: *E. coli* strain Rosetta (DE3) containing GST-AcWRKY75 plasmid before induction; 2: *E. coli* strain Rosetta (DE3) containing GST-AcWRKY75 plasmid after induction; 3: Purification of recombinant GST-AcWRKY75 protein.

**Fig. S3** AcWRKY75 and Ac4CL5 positively regulated phenolic accumulation in *Arabidopsis.* **A** Screening for T1 seedlings overexpressing *Ac4CL5* and *AcWRKY75* on resistance plate; Scale bar = 1 cm. **B**: PCR detection for T1 seedlings overexpressing *EV*, *Ac4CL5*, *AcWRKY75* and *AcMYB108*. **C-D** Phenotypes of *AcWRKY75 and Ac4CL5* overexpression *Arabidopsis* lines. Scale bar =1 cm.

**Fig. S4** AcMYB108 positively regulated phenolic accumulation in *Arabidopsis*. **A** Screening for T1 seedlings overexpressing *AcMYB108* on resistance plate; Scale bar = 1 cm. **B** Phenotypes of *AcMYB108* overexpression *Arabidopsis* lines. Scale bar =1 cm. **C** Expression of *AcMYB108* genes. Data are given as mean ± SD (n=3). Significance of differences is indicated by letters above the bars (*P*<0.05).

**Fig. S5** Purification of the recombinant GST-AcMYB108 protein used for the EMSA assay. Marker: protein marker; 1: *E. coli* strain Rosetta (DE3) containing GST-AcMYB108 plasmid before induction; 2: *E. coli* strain Rosetta (DE3) containing GST-AcMYB108 plasmid after induction; 3: Purification of recombinant GST-AcMYB108 protein.

**Table S1.** Sequences of primers used this study.

| Assay | Gene name | Primer name | Primer sequence |
| --- | --- | --- | --- |
| qRT-PCR | *AcActin* | *AcActin-F* | CTGAGGAGCACCCTGTCTTG |
|  |  | *AcActin-R* | GCATAGAGGGAGAGCACAGC |
|  | *AcWRKY75* | *AcWRKY75-F* | GAGAGGCGAGTTAGCGAAGG |
|  |  | *AcWRKY75-R* | CGTCGAGGATGTCGACTTGG |
|  | *AcMYB108* | *AcMYB108-F* | GCGGTAACATCACCCCAGAA |
|  |  | *AcMYB108-R* | TCGCGTGTTTTTGCACTCTG |
|  | *AcC4H* | *AcC4H -F* | GACCTGCAATCCAGAAGGCT |
|  |  | *AcC4H -R* | CGTTCTTCTCCGCTTCGAGT |
|  | *Ac4CL5* | *Ac4CL5-F* | GCTCGTCGAGAGGTACAAGG |
|  |  | *Ac4CL5-R* | TATCACGAGCCGAACCGATG |
|  | *AcHCT4* | *AcHCT4-F* | TACACGAGCGCGATCTCAAA |
|  |  | *AcHCT4-R* | CTACCGCATACGTGGCTGAT |
|  | *AcC3H* | *AcC3H-F* | AAGAACACCTCTCCAGCGTG |
|  |  | *AcC3H-R* | TCCTTGCTCGTCAATCACCC |
|  | *AcCSE* | *AcCSE-F* | CTTCACCCAGTCCTTCCACC |
|  |  | *AcCSE-R* | GTAGGCCATGGCGATCTTCT |
|  | *AtC4H* | *AtC4H-F* | GGAAACGTTTGCAATTGATGATGT |
|  |  | *AtC4H-R* | GCAATTCCCCACTCGATAGACCAC |
|  | *At4CL* | *At4CL-F* | CTAATGCCAAACTCGGTCAGGGATAC |
|  |  | *At4CL-R* | CTCTTGTAAAACACAACCTGTTTCGAC |
|  | *AtHCT* | *AtHCT-F* | CTTCTCGTTTTGCAGGTGACTTTC |
|  |  | *AtHCT-R* | GTATTCTCAGGTCCTGATTTAG |
|  | *AtC3H* | *AtC3H-F* | CATTGGTCTTCTATGGGATATG |
| Assay | Gene name | Primer name | Primer sequence |
|  |  | *AtC3H-R* | CTTCGGTGAGGTAGCATTAGA |
|  | *AtCSE* | *AtCSE-F* | CCGATGGTATCCGCTGCTACATGGGTG |
|  |  | *AtCSE-R* | TTGTTCCCACTCTAGGCTTCCCTGTATATCTTTG |
|  | *AtActin* | *AtActin-F* | CCTCAGCACATTCCAGCAGATGT |
|  |  | *AtActin-R* | TGGGACTAAAACGCAAAACGAAAG |
| Subcellular localization | *AcWRKY75-GFP* | *AcWRKY75-GFP-F* | CCAAATCGACTCTAGTCTAGAATGGAGAATTATTCCATACTATTCCCA |
|  |  | *AcWRKY75-GFP-R* | CCCGGGCCCCTGCAGAAGCTTTAAAGCTAGGATAAAGTTGCATTTGAT |
|  | *AcMYB108-GFP* | *AcMYB108-GFP-F* | CCAAATCGACTCTAGTCTAGAATGGAGGACCATGAAAGGGTC |
|  |  | *AcMYB108-GFP-R* | CCCGGGCCCCTGCAGAAGCTTTTTGGTGCATGAACAGTATGTCC |
| Overexpression assay | *AcWRKY75-GFP* | *AcWRKY75-GFP-F* | CCAAATCGACTCTAGTCTAGAATGGAGAATTATTCCATACTATTCCCA |
|  |  | *AcWRKY75-GFP-R* | CCCGGGCCCCTGCAGAAGCTTTAAAGCTAGGATAAAGTTGCATTTGAT |
|  | *AcMYB108-GFP* | *AcMYB108-GFP-F* | CCAAATCGACTCTAGTCTAGAATGGAGGACCATGAAAGGGTC |
|  |  | *AcMYB108-GFP-R* | CCCGGGCCCCTGCAGAAGCTTTTTGGTGCATGAACAGTATGTCC |
|  | *Ac4CL5-GFP* | *AcMYB108-GFP-F* | CCAAATCGACTCTAGTCTAGAATGCTGACCCACCGGGGG |
|  |  | *AcMYB108-GFP-R* | CCCGGGCCCCTGCAGAAGCTTTGTGCCGTCCGTTGGGAT |
|  | *35S::pSoup1300-GFP* | *35S::pSoup1300-GFP-For* | GCCATTTCGCCTTTTCAG |
|  |  | *35S::pSoup1300-GFP- Rev* | GCTGAACTTGTGGCCGTTTACGTC |
| VIGS | *V-Acwrky75* | *V-Acwrky75 -For* | AAGGTTACCGAATTCTCTAGAATGGAGAATTATTCCATACTATTCC |
|  |  | *V-Acwrky75-GFP-Rev* | TGTCTTCGGGACATGCCCGGGCGACCTTCGCTAACTCG |
|  | *35S::PTRV2* | *35S::PTRV2-For* | TGGGAGATGATACGCTGTT |
|  |  | *35S::PTRV2-Rev* | CCTAAAACTTCAGACACG |
| Assay | Gene name | Primer name | Primer sequence |
| Dua-luciferase reporter assay | *AcWRKY75-62-SK* | *AcWRKY75-62-SK-For* | AGAACTAGTGGATCCCCCGGGATGGAGAATTATTCCATACTATTCC- |
|  |  | *AcWRKY75-62-SK-Rev* | GATAAGCTTGATATCGAATTCAAAGCTAGGATAAAGTTGC |
|  | *AcMYB108-62-SK* | *AcMYB108-62-SK-For* | AGAACTAGTGGATCCCCCGGGATGGAGGACCATGAAAGGGTCG |
|  |  | *AcMYB108-62-SK-Rev* | GATAAGCTTGATATCGAATTCTTGGTGCATGAACAGTATGTCC |
|  | *0800-Ac4CL5pro* | *0800-Ac4CL5pro-For* | GTCGACGGTATCGATAAGCTTTTCTCAAAGCGCTGTGAGCT |
|  |  | *0800-Ac4CL5pro-Rev* | AGAACTAGTGGATCCCCCGGGCACCCCCTTCGGCAGC |
|  | *0800- AcWRKY75pro* | *0800- AcWRKY75pro-For* | GTCGACGGTATCGATAAGCTTAGTAGCCGGACTCGTAATGT |
|  |  | *0800-AcWRKY75pro-Rev* | AGAACTAGTGGATCCCCCGGGCTCCATAGAGGTGTGCAAGC |
| Y1H | *AcWRKY75-pJG4-5* | *AcWRKY75-pJG4-5*-*For* | AGATTATGCCTCTCCCGAATTCATGGAGAATTATTCCATACTATTCC |
|  |  | *AcWRKY75-pJG4-5*-*Rev* | AGAAGTCCAAAGCTTCTCGAGTTAAAAGCTAGGATAAAGTTGC |
|  | *AcMYB108-pJG4-5* | *AcMYB108-pJG4-5*-*For* | AGATTATGCCTCTCCCGAATTCATGGAGGACCATGAAAGGGTCG |
|  |  | *AcMYB108-pJG4-5*-*Rev* | AGAAGTCCAAAGCTTCTCGAGTTATTGGTGCATGAACAGTATGTCC- |
|  | *pLacZi2µ-Ac4CL5pro* | *pLacZi2µ-Ac4CL5pro-For* | CTTTGATATTGGATCGAATTCTTCTCAAAGCGCTGTGAGCT |
|  |  | *pLacZi2µ-Ac4CL5pro-Rev* | ATACAGAGCACATGCCTCGAGGGTCAGCATCACCCCCTTC |
|  | *pLacZi2µ-AcC4Hpro* | *pLacZi2µ-AcC4Hpro-For* | CTTTGATATTGGATCGAATTCCTCCTGCTTTCCATGCCCTT |
|  |  | *pLacZi2µ-AcC4Hpro-Rev* | ATACAGAGCACATGCCTCGAGGATAGCCATTTTCATCAATT |
|  | *pLacZi2µ-AcCSEpro* | *pLacZi2µ-AcCSEpro-For* | CTTTGATATTGGATCGAATTCAGAGGAAGTGCCAGGAAGTG |
|  |  | *pLacZi2µ-AcCSEpro-Rev* | ATACAGAGCACATGCCTCGAGGGGATGAGCCATGAGGAGAA |
|  | *pLacZi2µ-AcWRKY75pro* | *pLacZi2µ-AcWRKY75pro-For* | CTTTGATATTGGATCGAATTCGCCTCCAAACCCTTCCATTC |
|  |  | *pLacZi2µ- AcWRKY75pro-Rev* | ATACAGAGCACATGCCTCGAGCTCCATAGAGGTGTGCAAGC |
| EMSA | *GST-AcWRKY75* | *GST- AcWRKY75-F* | GATCTGGTTCCGCGTGGATCCATGGAGAATTATTCCATACTATTCC |
|  |  | *GST- AcWRKY75-R* | CTCTGAGTCGACCCGGGAATTCAAAGCTAGGATAAAGTTGC |
|  | *GST- AcMYB108* | *GST- AcMYB108 -For* | GATCTGGTTCCGCGTGGATCCATGGAGGACCATGAAAGGGTCG |
|  |  | *GST- AcMYB108-Rev* | TTACTCGAGTCGACCCGGGAATTCTTGGTGCATGAACAGTATGTCC |
|  | *EMSA- AcWRKY75pro* | *EMSA- AcWRKY75pro-For* | gcaggtaaaatataaatcaacagcgtaaattgctgagact |
| Assay | Gene name | Primer name | Primer sequence |
|  |  | *EMSA- AcWRKY75pro-Rev* | agtctcagcaatttacgctgttgatttatattttacctgc |
|  | *EMSA-mAcWRKY75pro* | *EMSA-mAcWRKY75pro-For* | gcaggtaaaatataaatAAAAAAcgtaaattgctgagact |
|  |  | *EMSA-mAcWRKY75pro-Rev* | agtctcagcaatttacgAAAAAAatttatattttacctgc |
|  | *EMSA-Ac4CL5pro* | *EMSA-Ac4CL5pro-For* | TCATGCGGACGTCGGTCCAGTTGAACCAGTCCGCCCTTCC |
|  |  | *EMSA- Ac4CL5pro-Rev* | GGAAGGGCGGACTGGTTCAACTGGACCGACGTCCGCATGA |
|  |  | *EMSA-mAc4CL5pro-For* | TCATGCGGACGTCGGTCAAAAAAAACCAGTCCGCCCTTCC |
|  |  | *EMSA-mAc4CL5pro-Rev* | GGAAGGGCGGACTGGTTAAAAAAGACCGACGTCCGCATGA |
|  | *EMSA-Ac4CL5pro* | *EMSA-Ac4CL5pro-For* | GTATATACGATCAACAGGTCAAGGCCCTCGGCATTCCGAT |
|  |  | *EMSA- Ac4CL5pro-Rev* | ATCGGAATGCCGAGGGCCTTGACCTGTTGATCGTATATAC |
|  |  | *EMSA-mAc4CL5pro-For* | GTATATACGATCAACAAAAAAAGGCCCTCGGCATTCCGAT |
|  |  | *EMSA-mAc4CL5pro-Rev* | ATCGGAATGCCGAGGGCCAAAAAATGTTGATCGTATATAC |
| GUS | *pCAMBIA1391- AcWRKY75* | *pCAMBIA1391- AcWRKY75-For* | TGGGCCCGGCGCGCCAAGCTTAGTAGCCGGACTCGTAATGT |
|  |  | *pCAMBIA1391- AcWRKY75-Rev* | CGGGGATCCGTCGACCTGCAGCTCCATAGAGGTGTGCAAGC |
|  | *pCAMBIA1391- AcWRKY75-1* | *pCAMBIA1391-AcWRKY75-1-For* | TGGGCCCGGCGCGCCAAGCTTAGTAGCCGGACTCGTAATGT |
|  |  | *pCAMBIA1391-AcWRKY75-1-Rev* | CGGGGATCCGTCGACCTGCAGCTCACCTCCTGCTCCATTTC |
|  | *pCAMBIA1391-AcWRKY75-2* | *pCAMBIA1391-AcWRKY75-2-For* | TGGGCCCGGCGCGCCAAGCTTGAAATGGAGCAGGAGGTGAG |
|  |  | *pCAMBIA1391-AcWRKY75-2-Rev* | CGGGGATCCGTCGACCTGCAGCTCCATAGAGGTGTGCAAGC |
|  | *pCAMBIA1391- Ac4CL5* | *pCAMBIA1391- Ac4CL5-For* | TGGGCCCGGCGCGCCAAGCTTTTCTCAAAGCGCTGT |
|  |  | *pCAMBIA1391- Ac4CL5-Rev* | CGGGGATCCGTCGACCTGCAGCACCCCCTTCGGCAG |
|  | *pCAMBIA1391- Ac4CL5-1* | *pCAMBIA1391- Ac4CL5-1-For* | TGGGCCCGGCGCGCCAAGCTTGGTAGGTGGCTCCGAACTT |
|  |  | *pCAMBIA1391- Ac4CL5-1-Rev* | CGGGGATCCGTCGACCTGCAGACCCCACGTCAACGTCCC |
| Assay | Gene name | Primer name | Primer sequence |
|  | *pCAMBIA1391- Ac4CL5-2* | *pCAMBIA1391- Ac4CL5-2-For* | TGGGCCCGGCGCGCCAAGCTTTTTCTGTTGGAAAAGAGCTATT |
|  |  | *pCAMBIA1391- Ac4CL5-2-Rev* | CGGGGATCCGTCGACCTGCAGGTCGCCGGTGGTTACGATC |
|  | *pCAMBIA1391- Ac4CL5-3* | *pCAMBIA1391- Ac4CL5-3-For* | TGGGCCCGGCGCGCCAAGCTTCGATCGTAACCACCGGCGAC |
|  |  | *pCAMBIA1391- Ac4CL5-3-Rev* | CGGGGATCCGTCGACCTGCAGCTGTCGTCCCCGATGAGT |
|  | *pCAMBIA1391- Ac4CL5-4* | *pCAMBIA1391- Ac4CL5-4-For* | TGGGCCCGGCGCGCCAAGCTTGCTCCCCGACAAATACCGAA |
|  |  | *pCAMBIA1391- Ac4CL5-4-Rev* | CGGGGATCCGTCGACCTGCAGGGTTGGAGCGAGTTTTCTCC |
|  | *pCAMBIA1391- AcMYB108* | *pCAMBIA1391- AcMYB108-For* | TGGGCCCGGCGCGCCAAGCTTGTCTGTATGCCCTTTGGGCA |
|  |  | *pCAMBIA1391- AcMYB108-Rev* | CGGGGATCCGTCGACCTGCAGTCCTCCATGAGAAAAGGGTGGG |
|  | *pCAMBIA1391-AcMYB108-1* | *pCAMBIA1391-AcMYB108-1-For* | TGGGCCCGGCGCGCCAAGCTTGTCTGTATGCCCTTTGGGCA |
|  |  | *pCAMBIA1391-AcMYB108-1-Rev* | CGGGGATCCGTCGACCTGCAGCTTGTCCCCAAAGTTTGAGC |
|  | *pCAMBIA1391-AcMYB108-2* | *pCAMBIA1391-AcMYB108-2-For* | TGGGCCCGGCGCGCCAAGCTTGCTCAAACTTTGGGGACAAG |
|  |  | *pCAMBIA1391-AcMYB108-2-Rev* | CGGGGATCCGTCGACCTGCAGTGCTCGTGTCTGTGTGTGTG |
|  | *pCAMBIA1391-AcMYB108-3* | *pCAMBIA1391-AcMYB108-3-For* | TGGGCCCGGCGCGCCAAGCTTCACACACACAGACACGAGCA |
|  |  | *pCAMBIA1391-AcMYB108-3-Rev* | CGGGGATCCGTCGACCTGCAGAAGCTAAAAGTACAGGGGCC |
|  | *pCAMBIA1391-AcMYB108-4* | *pCAMBIA1391-AcMYB108-4-For* | TGGGCCCGGCGCGCCAAGCTTAGGCCCCTGTACTTTTAGCTT |
|  |  | *pCAMBIA1391-AcMYB108-4-Rev* | CGGGGATCCGTCGACCTGCAGTCCTCCATGAGAAAAGGGTGGG |

**Table S2.** The top 10 differentially expressed TFs identified from ABA treatment *vs* Control.

| Gene ID | FPKM (ABA) | FPKM (Control) | Log2 (FC) | Gene description | TF family |
| --- | --- | --- | --- | --- | --- |
| LOC109718059 | 13.12±0.93 | 244.60±8.47 | -4.22 | transcription factor MYB108-like && Q9LDE1.1 RecName: Full=Transcription factor MYB108; AltName: Full=Myb-related protein 108; Short=AtMYB108; AltName: Full=Protein BOTRYTIS-SUSCEPTIBLE 1 && PF00249: Myb-like DNA-binding domain | Myb_DNA-binding |
| LOC109726197 | 10.10±0.75 | 173.08±26.76 | -4.10 | ethylene-responsive transcription factor ERF113-like && Q9FH54.1 RecName: Full=Ethylene-responsive transcription factor ERF114; AltName: Full=ERF bud enhancer && PF00847:AP2 domain | AP2 |
| LOC109726264 | 4.53±0.24 | 123.32±10.36 | -4.77 | probable WRKY transcription factor 75 && Q9FYA2.1 RecName: Full=Probable WRKY transcription factor 75; AltName: Full=WRKY DNA-binding protein 75 && PF03106: WRKY DNA -binding domain | WRKY |
| novel.420 | 4.11±0.93 | 66.66±2.36 | -4.02 | - && Q3EAL1.1 RecName: Full=Transcription repressor OFP6; AltName: Full=Ovate family protein 6; Short=AtOFP6 && - | - |
| LOC109724596 | 2.21±0.35 | 38.94±4.33 | -4.14 | WRKY transcription factor WRKY71-like && Q6QHD1.1 RecName: Full=WRKY transcription factor WRKY71; Short=OsWRKY71 && PF03106:WRKY DNA -binding domain | WRKY |
| Gene ID | FPKM (ABA) | FPKM (Control) | Log2 (FC) | Gene description | TF family |
| LOC109713189 | 0.43±0.14 | 36.51±1.36 | -6.39 | transcription factor JUNGBRUNNEN 1-like && Q9SK55.1 RecName: Full=Transcription factor JUNGBRUNNEN 1; AltName: Full=NAC domain-containing protein 42; Short=ANAC042 && PF02365: No apical meristem (NAM) protein | NAM |
| LOC109719592 | 1.70±0.31 | 35.68±2.59 | -4.39 | WRKY transcription factor WRKY24-like && Q6IEQ7.1 RecName: Full=WRKY transcription factor WRKY24; Short=OsWRKY24 && PF03106: WRKY DNA -binding domain | WRKY |
| LOC109713270 | 0.11±0.04 | 18.10±3.65 | -7.32 | ethylene-responsive transcription factor ERF096-like && P93822.1 RecName: Full=Ethylene-responsive transcription factor 14; Short=AtERF14; AltName: Full=Ethylene-responsive element-binding factor 14; Short=EREBP-14 && PF00847:AP2 domain | AP2 |
| LOC109708800 | 1.03±0.14 | 20.64±6.44 | -4.33 | myb-related protein 330-like && O22264.1 RecName: Full=Transcription factor MYB12; AltName: Full=Myb-related protein 12; Short=AtMYB12 && PF00249: Myb-like DNA-binding domain | Myb_DNA-binding |
| LOC109716989 | 1.08±0.12 | 20.08±1.64 | -4.21 | probable WRKY transcription factor 71%2C transcript variant X1 && Q93WV4.1 RecName: Full=Probable WRKY transcription factor 71; AltName: Full=WRKY DNA-binding protein 71 && PF03106: WRKY DNA -binding domain | WRKY |

**Table S3.** The top 10 differentially expressed TFs identified from ABA treatment *vs* GA_3_.

| Gene ID | FPKM (ABA) | FPKM (GA_3_) | Log2 (FC) | Gene description | TF family |
| --- | --- | --- | --- | --- | --- |
| LOC109718059 | 13.12±0.93 | 200.64±9.78 | -3.94 | transcription factor MYB108-like && Q9LDE1.1 RecName: Full=Transcription factor MYB108; AltName: Full=Myb-related protein 108; Short=AtMYB108; AltName: Full=Protein BOTRYTIS-SUSCEPTIBLE 1 && PF00249:Myb-like DNA-binding domain | Myb_DNA-binding |
| LOC109726197 | 10.10±0.75 | 165.89±10.04 | -4.04 | ethylene-responsive transcription factor ERF113-like && Q9FH54.1 RecName: Full=Ethylene-responsive transcription factor ERF114; AltName: Full=ERF bud enhancer && PF00847:AP2 domain | AP2 |
| LOC109726264 | 4.53±0.24 | 149.98±3.62 | -5.06 | probable WRKY transcription factor 75 && Q9FYA2.1 RecName: Full=Probable WRKY transcription factor 75; AltName: Full=WRKY DNA-binding protein 75 && PF03106:WRKY DNA -binding domain | WRKY |
| LOC109713641 | 11.88±0.17 | 140.55±10.27 | -3.57 | ethylene-responsive transcription factor 2-like && O80337.2 RecName: Full=Ethylene-responsive transcription factor 1A; Short=AtERF1A; AltName: Full=Ethylene-responsive element-binding factor 1A; Short=EREBP-1A && PF00847:AP2 domain | AP2 |
| LOC109713270 | 0.11±0.04 | 81.65±8.20 | -9.50 | ethylene-responsive transcription factor ERF096-like && P93822.1 RecName: Full=Ethylene-responsive transcription factor 14; Short=AtERF14; AltName: Full=Ethylene-responsive element-binding factor 14; Short=EREBP-14 && PF00847:AP2 domain | AP2 |
| Gene ID | FPKM (ABA) | FPKM (GA_3_) | Log2 (FC) | Gene description | TF family |
| LOC109713189 | 0.43±0.14 | 58.72±1.78 | -7.08 | transcription factor JUNGBRUNNEN 1-like && Q9SK55.1 RecName: Full=Transcription factor JUNGBRUNNEN 1; AltName: Full=NAC domain-containing protein 42; Short=ANAC042 && PF02365:No apical meristem (NAM) protein | NAM |
| LOC109724596 | 2.21±0.35 | 45.94±4.74 | -4.39 | WRKY transcription factor WRKY71-like && Q6QHD1.1 RecName: Full=WRKY transcription factor WRKY71; Short=OsWRKY71 && PF03106:WRKY DNA -binding domain | WRKY |
| LOC109719592 | 1.70±0.31 | 43.58±1.00 | -4.68 | WRKY transcription factor WRKY24-like && Q6IEQ7.1 RecName: Full=WRKY transcription factor WRKY24; Short=OsWRKY24 && PF03106:WRKY DNA -binding domain | WRKY |
| LOC109724369 | 0.23±0.18 | 42.13±0.81 | -7.51 | ethylene-responsive transcription factor ERF071 && O22259.1 RecName: Full=Ethylene-responsive transcription factor ERF071 && PF00847:AP2 domain | AP2 |
| LOC109708800 | 1.03±0.14 | 32.10±2.08 | -4.97 | myb-related protein 330-like && O22264.1 RecName: Full=Transcription factor MYB12; AltName: Full=Myb-related protein 12; Short=AtMYB12 && PF00249:Myb-like DNA-binding domain | Myb_DNA-binding |
